# Meta-analysis of mesenchymal stem cell therapy for intrauterine adhesions: a comprehensive consideration of efficacy and safety

**DOI:** 10.3389/fbioe.2025.1619778

**Published:** 2025-08-13

**Authors:** Jiawei Gao, Xinyue Zhou, Nan Jiang, Yongjuan Zhang, Jianpeng Han, Tianyu Jia, Yanbiao Jiang, Xiaoling Ma, Haofei Shen

**Affiliations:** ^1^ The First Clinical Medical College of Lanzhou University, Lanzhou, China; ^2^ Reproductive Medicine Center, The First Hospital of Lanzhou University, Lanzhou, China; ^3^ Clinical Research Center for Reproductive Diseases of Gansu Province, Lanzhou, China

**Keywords:** IUA, stem cell therapy, meta-analysis trial registration, safety, mesenchymal stem cells (MSCs)

## Abstract

**Background:**

Intrauterine adhesions (IUA), a common gynecological condition, often result from endometrial injury and fibrosis. Traditional therapies like hysteroscopic adhesiolysis and hormone therapy show limited efficacy in endometrial repair and high recurrence rates. Stem cell therapy, particularly using mesenchymal stem cells (MSCs), has emerged as a promising alternative. This meta-analysis evaluates the efficacy and safety of MSCs therapy for IUA.

**Methods:**

We systematically searched Embase, MEDLINE, and the Cochrane Library up to January 2025. Using random or fixed-effects models, we analyzed outcomes including pregnancy rates, endometrial thickness, menstrual improvement, and safety indicators. Subgroup analyses were performed based on stem cell sources and transplantation methods.

**Results:**

Twelve studies with 233 patients were included. Stem cell therapy significantly improved menstrual outcomes (RR = 34.54, 95%CI: [15.07–79.18]), pregnancy rates (RR = 21.86, 95%CI: [8.60–55.56]), live birth rates (RR = 18.00, 95%CI: [6.67–48.55]), and endometrial thickness (MD = 2.28mm, 95%CI: [1.60–2.96]). Subgroup analyses indicated that adipose-derived (MD = 3.34 mm) and menstrual blood-derived (MD = 3.62 mm) stem cells exhibited the highest efficacy. Safety data indicated mild abdominal pain in 5.15% and abnormal blood indices in 4.29% of patients, with no severe complications.

**Conclusion:**

Stem cell therapy may improve the reproductive prognosis of patients with IUA. Autologous adipose or menstrual blood-derived stem cells via local injection are cautiously recommended. Further large-scale RCTs are needed to confirm long-term safety and optimal treatment protocols.

**Trial registration:**

The review protocol is registered on PROSPERO with registration number CRD42025643871, and no modifications were made to the information provided at registration.

**Systematic Review Registration:**

https://www.crd.york.ac.uk/prospero/, identifier CRD420256438.

## 1 Introduction

### 1.1 Overview of IUA

Intrauterine adhesions (IUA), also known as Asherman’s syndrome (AS), is a intrauterine common disorder usually caused by fibrotic regeneration of the uterine lining following endometrial injury (Cao et al., 2018a). This injury may originate from intrinsic factors, or it may be caused by disease (endometritis or congenital dysplasia) or medical intervention (such as surgery, hormone therapy, curettage or radiation therapy). Damage to the lining of the uterus and scarring prevents normal menstrual cycles and embryo implantation, severely affecting a woman’s ability to have children, and is one of the common causes of female infertility (Rodríguez-Eguren et al., 2024). Currently, traditional treatments for IUA include surgical separation of adhesions and hormonal therapy, but these methods have certain limitations. For example, hysteroscopic adhesiolysis is widely recommended as an effective treatment, but the prognosis and recurrence rate remain poor in severe cases (Bai et al., 2020). Current clinical procedures have not improved the prognosis of patients with moderate to severe IUA with recurrence rates of 23%–50% (Xia et al., 2022). Hormonal therapy is also often unsatisfactory in improving endometrial regeneration (Cittadini et al., 2022).

### 1.2 Application of stem cell therapy

Given the limitations of traditional treatments, stem cell therapy has shown potential as an emerging treatment option for IUA (Hu et al., 2024). Stem cell transplantation is recognized as a potential alternative therapy to facilitate the recovery process (Gan et al., 2017). MSCs and their exosomes are considered as a candidate therapy for repairing damaged endometrium (Mansouri-Kivaj et al., 2023). The possible mechanisms of stem cell therapy for IUA are: first, direct differentiation into endometrial cells (Zheng et al., 2020), involved in endometrial repair and regeneration. Secondly, they secrete growth factors (e.g., transforming growth factor β) through paracrine action to promote the repair of damaged endometrium. These factors can stimulate the proliferation and differentiation of endometrial cells and reduce fibrosis, thus improving the endometrial environment and promoting angiogenesis and cell proliferation (Chen et al., 2024). In addition, stem cells can reduce fibrotic areas of the endometrium, which is essential for restoring the normal structure and function of the endometrium (Zhang et al., 2018). By reducing fibrosis, stem cells help to improve the endometrial receptivity, thereby increasing the likelihood of embryo implantation.

Current studies have shown that MSCs play an important role in repairing the damaged endometrium, including bone marrow mesenchymal stem cells (BMSCs) (Yuan et al., 2022), umbilical cord blood mesenchymal stem cells (UC-MSCs) (Cao et al., 2018a), amniotic membrane mesenchymal stem cells (hAMSCs) (Gan et al., 2017), and adipose-derived stem cells (ADSCs) (Zhao et al., 2021). These stem cells come from different sources, but all show the ability to promote endometrial repair and regeneration. Studies have explored the clinical applications of stem cell therapy, including the use of stem cells in combination with biomaterials such as collagen scaffolds to enhance therapeutic effects. MSCs, in particular, have been widely used in the clinic due to their low immunogenicity (Chen et al., 2021). However, stem cell therapies are subject to certain limitations in clinical applications, including problems with precise induction of differentiation, tumor formation and unclear molecular mechanisms (Zhou et al., 2022). The main challenges of stem cell therapy include loss of stem cell identity, immunogenicity, low retention and survival (Xi et al., 2022).

In summary, stem cell therapy offers a promising new approach to treating IUA, especially when conventional treatments have limited effectiveness. However, the effectiveness and safety of stem cell therapy still need to be verified by further research and clinical trials.

## 2 Materials and methods

### 2.1 Literature search strategy

Databases including Embase, MEDLINE and Cochrane Library were searched up to January 2025. The search terms included “Stem Cell “ and “IUA “ and their related synonyms and proximity, and combinations of Boolean logic operators (e.g., AND, OR) were used. At the same time, references to the incorporated literature were manually searched to ensure comprehensive access to relevant studies. The detailed search strategy is shown in Table 1. The review protocol was registered on PROSPERO under registration number CRD42025643871, and the information provided at the time of registration was not modified in any way.

**TABLE 1:** Search strategy.

Cochrany library 2025-01-23	Searches	Results
#1	MeSH descriptor: [Stem Cell Transplantation] explode all trees	3,628
#2	MeSH descriptor: [Bone Marrow Cells] explode all trees	2,172
#3	MeSH descriptor: [Stem Cells] explode all trees	1,354
#4	MeSH descriptor: [Cell Transplantation] this term only	3,792
#5	MeSH descriptor: [Bone Marrow Transplantation] this term only	1,696
#6	MeSH descriptor: [Stromal Cells] explode all trees	451
#7	((stem or haematopoietic or hematopoietic or haematopoetic or hematopoetic or hemopoietic or haemopoietic or progenitor or precursor or bone marrow or mononuclear or “adipose tissue” or mesenchymal or stromal or autologous or allogeneic or allogenic) next/2 cell*)	28,567
#8	“cell transplantation”:so or “stem cell”:so or “bone marrow transplantation”:so	1,431
#9	(autologous next/3 transplant*) or cell* next therap*”	7,769
#10	((cell*) near/3 (autologous or transplant* or autotransplant* or allotransplant* or graft* or implant*))	16,313
#11	#1 or #2 or #3 or #4 or #5 or #6 or #7 or #8 or #9 or #10	34,353
#12	(intrauterine or uterine) next/3 (adhesio* or synechia*) or IUA	743
#13	asherma* next/3 syndrom*	79
#14	#12 or #13	779
#15	#11 and #14	22

MEDLINE Ovid 2025-01-23		
#1	exp Stem Cell Transplantation/ or Bone Marrow Transplantation/ or Cell Transplantation/ or exp Stem Cells/ or Bone Marrow Cells/ or exp Stromal Cells/	439,725
#2	((stem or haematopoietic or hematopoietic or haematopoetic or hematopoetic or hemopoietic or haemopoietic or progenitor or precursor or bone marrow or mononuclear or adipose tissue or mesenchymal or stromal or autologous or allogeneic or allogenic) adj2 cell*).ti,ab.	643,744
#3	(cell transplantation or stem cell* or bone marrow transplantation).jn.	438,88
#4	((autologous adj3 transplant*) or cell* therap*).tw.	66,962
#5	(cell* adj3 (autologous or transplant* or autotransplant* or allotransplant* or graft* or implant*)).ti,ab.	148,167
#6	1 or 2 or 3 or 4 or 5	822,813
#7	(((intrauterine or uterine) adj3 (adhesio* or synechia*)) or IUA).tw.	1,860
#8	(asherma* adj3 syndrom*).tw.	463
#9	7 or 8	2,124
#10	6 and 9	308

Embase Ovid 2025-01-23		
#1	exp Stem Cell Transplantation/ or exp Bone Marrow Transplantation/ or exp Stem Cell/ or Bone Marrow Cell/	786,020
#2	((stem or haematopoietic or hematopoietic or haematopoetic or hematopoetic or hemopoietic or haemopoietic or progenitor or precursor or bone marrow or mononuclear or adipose tissue or mesenchymal or stromal or autologous or allogeneic or allogenic) adj2 cell*).ti,ab.	920,181
#3	(cell transplantation or stem cell* or bone marrow transplantation).jn.	58,162
#4	((autologous adj3 transplant*) or cell* therap*).tw.	113,103
#5	((cell* or myoblast*) adj3 (autologous or transplant* or autotransplant* or allotransplant* or graft* or implant*)).ti,ab.	251,928
#6	1 or 2 or 3 or 4 or 5	1,215,551
#7	exp uterus synechia/	2,588
#8	(((intrauterine or uterine) adj3 (adhesio* or synechia*)) or IUA).tw.	2,589
#9	(asherma* adj3 syndrom*).tw.	774
#10	7 or 8 or 9	3,881
#11	6 and 10	443

### 2.2 Inclusion and exclusion criteria

Literature was screened and cross-checked independently by two researchers (Jiawei Gao and Xinyue Zhou). In case of disagreement, discussion was held first, and if no agreement could be reached, the third investigator (Haofei Shen) made the final decision after group discussion. The inclusion criteria were as follows: (1) Participants: patients diagnosed with IUA (Asherman syndrome). Clinical manifestations may include partial or complete occlusion of the uterine cavity, decreased menstrual flow or even amenorrhea, infertility and cyclic abdominal pain; (2) Intervention: patients received treatment with autologous or allogeneic MSCs. Including stem cells from various sources such as umbilical cord, umbilical cord blood, adipose, menstrual blood, endometrium, bone marrow, etc.; (3) The study was written or published in English; (4) The outcome metrics consisted of at least one of the pregnancy rate, endometrial thickness, menstrual improvement, and/or adverse events reported. (5) The study adopted a self-controlled clinical trial design. Efficacy was assessed by comparing the metrics before and after treatment of patients with MSCs. The exclusion criteria were (1) incompatible study types, such as reviews, case reports, Meta-analyses, systematic evaluations, guidelines, letters, conference abstracts, unpublished studies, and editorial comments, etc.; (2) ineligible study subjects, excluding animal studies and *in vitro* experimental studies; (3) irrelevant study topics, i.e., not a study that explored the relationship between stem cell therapy and IUA; (4) non-English articles; (5) Data were not accessible, including cases such as no response to contact with the authors.

### 2.3 Data extraction

For each study that met the inclusion criteria, two investigators (Jiawei Gao and Xinyue Zhou) independently performed information extraction and cross-checking, which included the following data: first author, year of publication, type of study, country of the first author, sample size, time frame of inclusion and age of the study subjects, type of stem cells, source of the samples (e.g., blood, bone marrow, umbilical cord, etc.), and outcome indicators.

### 2.4 Quality assessment

Quality assessment was independently conducted by two researchers (Jiang Nan and Han Jianpeng) with subsequent cross-verification. In case of disagreements, a discussion was held, and if unresolved, a third researcher (Shen Haofei) made the final decision following a group discussion. The ROBINS-I tool was employed to evaluate the included studies (Sterne et al., 2016).

### 2.5 Data processing and analysis

Meta-analysis was performed using revman 5.4 and stata 15 software. For dichotomous variables (e.g., pregnancy rate, menstrual improvement), Risk Ratio (RR) and its 95% Confidence Interval (CI) were used for analysis; for continuous variables (e.g., endometrial thickness), Mean Difference (MD) and its 95% CI were used for analysis. Heterogeneity of the included studies was assessed by the I^2^ statistic, and data were combined using a fixed-effects model if I^2^ ≤ 50% or a randomized effects model if I^2^ >50%. Only when more than 10 articles were included, meta-regression analysis was performed to explore the source of heterogeneity. Publication bias analysis was performed only when more than 10 papers were included in the Meta-analysis.

## 3 Results

### 3.1 Study selection process

**FIGURE 1:**
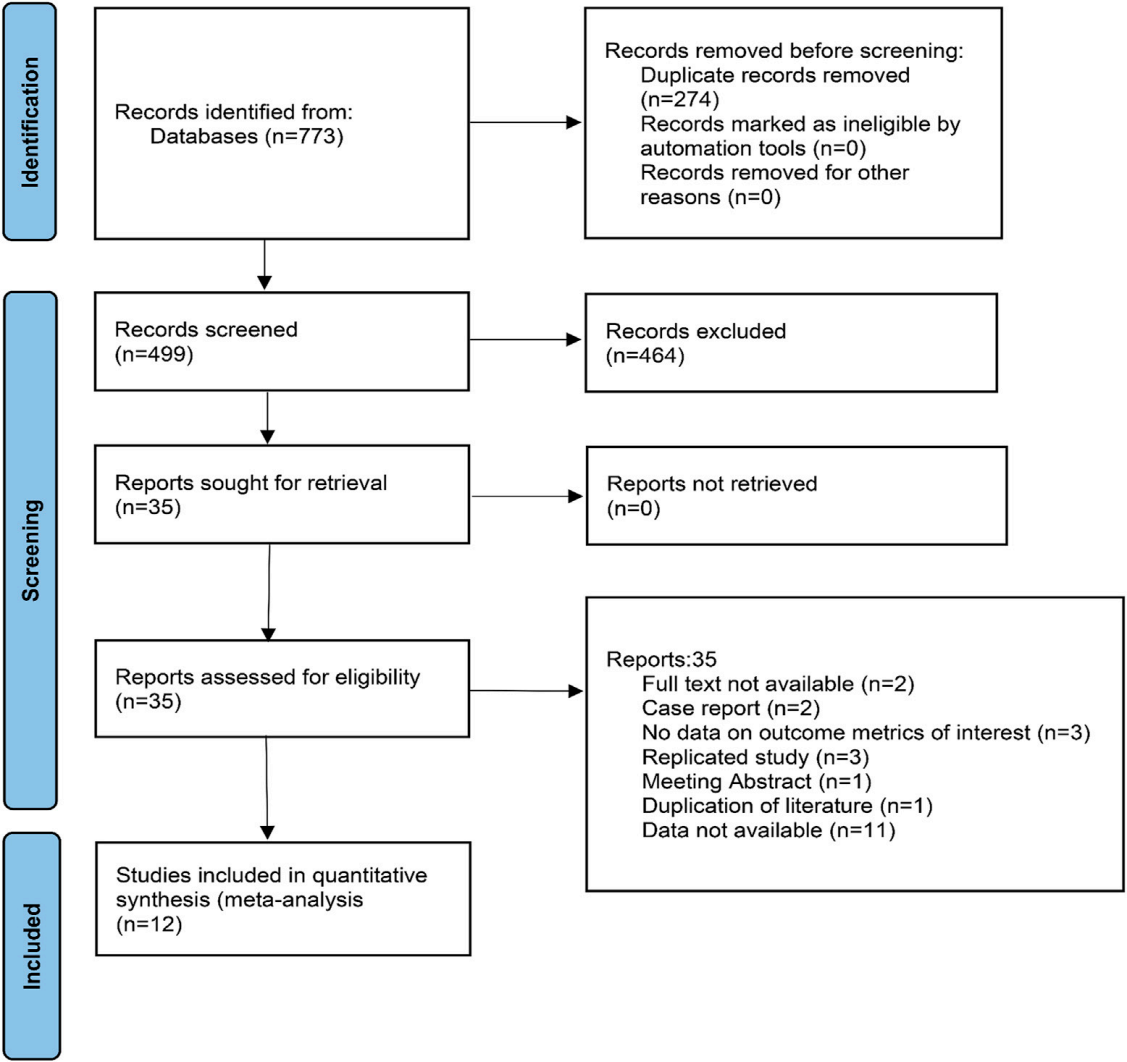
Flow diagram of the study selection process.

A total of 773 documents were retrieved from three electronic databases and other sources. After automatic removal of duplicate literature, 499 articles remained. By reading the titles and abstracts, 464 were excluded, with the main reasons including that the literature was a review, a commentary, an animal study, or not relevant to the study topic. The remaining 35 literature were read in full and 12 studies were finally included. The literature screening process is shown in Figure 1.

### 3.2 Characteristics of the included studies

We included 12 studies (233 patients). Countries such as Turkey, China, Mexico, South Korea, Spain, and India were involved, and the studies spanned from 2013 to 2024. The patients were all IUA patients with an age range of 22–45 years, and most of them had unsuccessful previous treatment attempts such as hysteroscopic adhesion release and hormonal therapy. Stem cell sources were diverse, including autologous bone marrow, umbilical cord, adipose tissue, and menstrual blood. The duration of follow-up varied, ranging from 1 week to 5 years Table 2 summarizes the detailed characteristics of the 12 articles.

**TABLE 2:** Results of basic information about the articles included.

Author +year	Country	Number	Period	Age	Prior repair attempts	Stem cells	Source of stem cells	Dose	Follow up
Arikan et al. (2003)	Turkey	20	2018-2020	36.6 ± 5.59	HRT	autologous bone marrow nucleated cells (aBMNC)	Autologous bone marrow	First injection:11.55 ± 4.7 × 108 Second injection: 6.85 ± 2.67 × 108	3, 6, 9 months
Cao et al. (2018)	China	26	No mention	27-42(35.1 ± 3.8)	Hysteroscopic adhesiolysis	allogeneic UC-MSC	allogenic, loaded onto a collagen scaffold	1 × 10^7^ (4.2 × 10^5^/cm^2^)	30 months
Hernandez-Melchor et al. (2024)	Mexico	21	2020-2022	40.0 ± 4.7	Hysteroscopic adhesiolysis	autologous ADSCs	Autologous Stromal Vascular Fraction (SVF) of adipose tissue	3ml	1 week
Huang et al. (2022)	China	10	2019-2020	29-40(33.9 ± 3.75)	Hysteroscope and drug treatment	hUC-MSCs	allogenic	2 ml (1*107/ml)	6 months
Lee et al. (2020)	Korea	5	2015-2017	36-43(39.2 ± 2.8)	Hysteroscopic adhesiolysis, HRT	autologous ADSCs	Autologous Stromal Vascular Fraction (SVF) of adipose tissue	4.6 ± 0.7 × 10^6^	23 months
Ma et al. (2020)	China	12	No mention	22-40(35.8 ± 3.6)	Adhesiolysis	autologous menSCs	Autologous menstrual blood	10 × 10^6^	No mention
Santamaria (2016)	Spain	11	No mention	30-45(38.0 ± 4.8)	Hysteroscope	autologous BMDSCs	autologous BMDSCs	123.56 × 10^6^ (42-200 × 10^6^)	6 months
Shi et al. (2021)	China	16	2019-2020	30-39(34.1 ± 3.6)	Hysteroscopic adhesiolysis	Allogeneic UC-MSC	allogenic, loaded onto a collagen scaffold	1 × 10^7^/mL (2mL)	27 months
Singh et al. (2020)	India	25	2013-2019	24-38(29.6 ± 4.1)	Hysteroscopic adhesiolysis,HRT	autologous BMNCs	autologous BMNCs	65.3 × 10^6^ ± 37.2 (19-200 × 10^6^)	3, 6, 9 months, 5years
Singh et al. (2014)	India	6	2013-2014	25-35(29.8 ± 3.4)	Hysteroscopic adhesiolysis,HRT	autologous BMNCs	autologous BMNCs	103.3 × 10^6^ ± 20.45	3, 6, 9 months
Tan et al. (2016)	China	7	No mention	20-40(33.7 ± 1.5)	Adhesiolysis、IUD、HRT	autologous menSCs	autologous menSCs	1 × 10^6^	3, 4, 6 months
Zhu et al. (2024)	China	74	2016-2020	31.3 ± 4.2	Hysteroscope	autologous bone marrow stem cells (BMSCs)	autologous bone marrow stem cells-scaffold	/	/

### 3.3 Methodological quality and risk of bias

Potential risk of bias was assessed using the ROBINS-I tool. As shown in Table 3, multiple key aspects of pre-intervention, intervention, and post-intervention were analyzed in each included study and assessed in multiple dimensions related to confounding bias, selection bias, intervention categorization bias, intervention implementation bias, missing data bias, outcome measurement bias, and selection bias in reporting outcomes. In addition, we performed a comprehensive assessment of the overall risk of bias for each study. As shown in the table, 11 (91.7%) of the 12 studies presented a high risk of confounding factor bias (D1). Participant selection selection bias (D2) was generally at medium risk, while intervention categorization bias (D3) showed low risk in 10 studies (83.3%). Notably, missing data bias (D5) was at low risk in five studies (41.7%) such as Arikan et al. (2003) but Singh et al. (2020) were at high risk in this area. Overall risk assessment showed that nine studies (75%) were at high risk of bias, 2 (16.7%) were at moderate risk, and only (Zhu et al., 2024a) met the overall low risk criteria.

**TABLE 3:** Quality assessment list of all studies.

Study	D1	D2	D3	D4	D5	D6	D7	OverAll
Arikan et al. (2023)	×	-	-	-	+	+	×	×
Cao et al. (2018)	×	-	-	+	+	-	+	×
Hernandez-Melchor et al. (2024)	-	-	+	+	+	-	+	-
Huang et al. (2022)	×	-	+	+	-	-	+	×
Lee et al. (2020)	×	-	+	+	-	-	+	-
Ma et al. (2020)	×	-	+	+	+	-	+	×
Santamaria (2016)	×	-	+	+	+	-	+	×
Zhang et al. (2020)	×	-	+	+	+	-	+	×
Singh et al. (2020)	-	-	+	+	×	-	+	×
Singh et al. (2014)	-	-	+	+	-	+	+	-
Tan et al. (2016)	×	-	+	+	+	-	+	×
Zhu et al. (2024)	+	+	+	+	+	+	+	+

D1: Bias due to confounding factors

D2: Bias due to participants' choices

D3: Bias in the classification of interventions

D4: Deviations from intended interventions

D5: Deviation due to missing data

D6: Bias in outcome measurement

D7: Bias in the selection of reporting results

OverAll: The overall risk of bias judgments is determined by the highest risk assigned to any single domain.

+: Low risk of bias

-: Moderate risk of bias

×: High risk of bias

### 3.4 Efficacy evaluation

#### 3.4.1 Menstrual improvement

**FIGURE 2:**
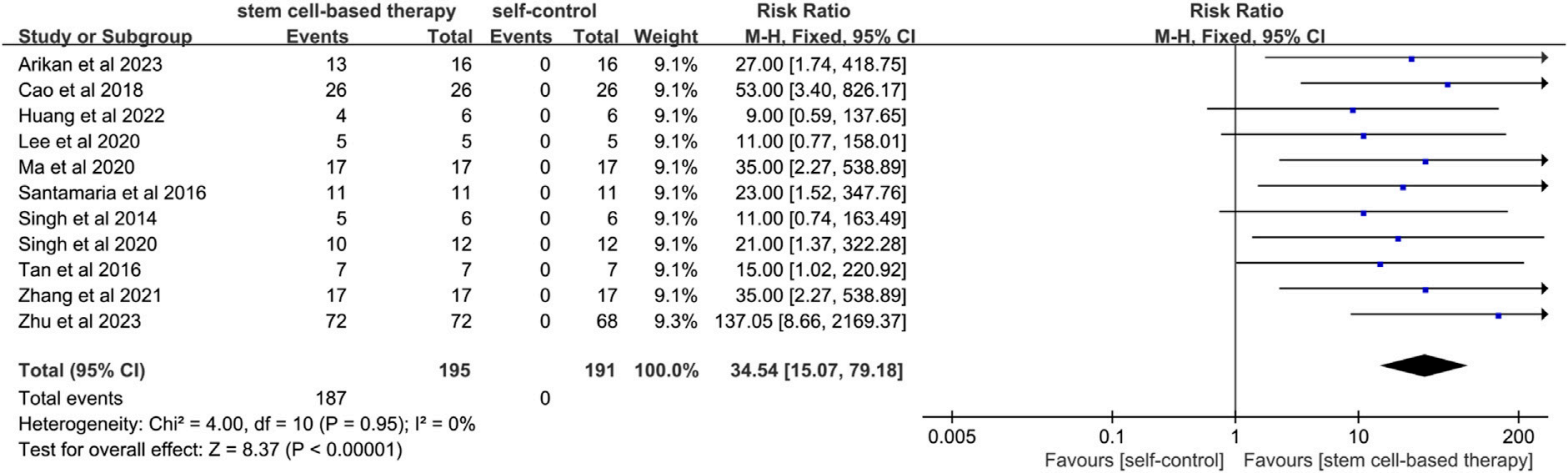
Forest plot of menstruation improvement in stem cell therapy for IUA.

A total of 11 studies mentioned menstrual improvement. (Figure 2). Menstrual improvement was assessed by comparing changes in patients' own menstrual flow before and after stem cell therapy for IUA. Since the statistical heterogeneity among studies was not significant (P = 0.95; I^2^ = 0%), a fixed-effects model was used and the results showed statistical significance (RR = 34.54, 95% CI: [15.07, 79.18]). This indicates a significant effect of stem cell therapy on menstrual improvement. The results of sensitivity analysis showed that the results of meta-analysis were still statistically significant after excluding studies of high risk of bias (RR = 53.80, 95% CI [10.86, 266.54]) (Supplementary Figure S1).

#### 3.4.2 Clinical pregnancy rate

**FIGURE 3:**
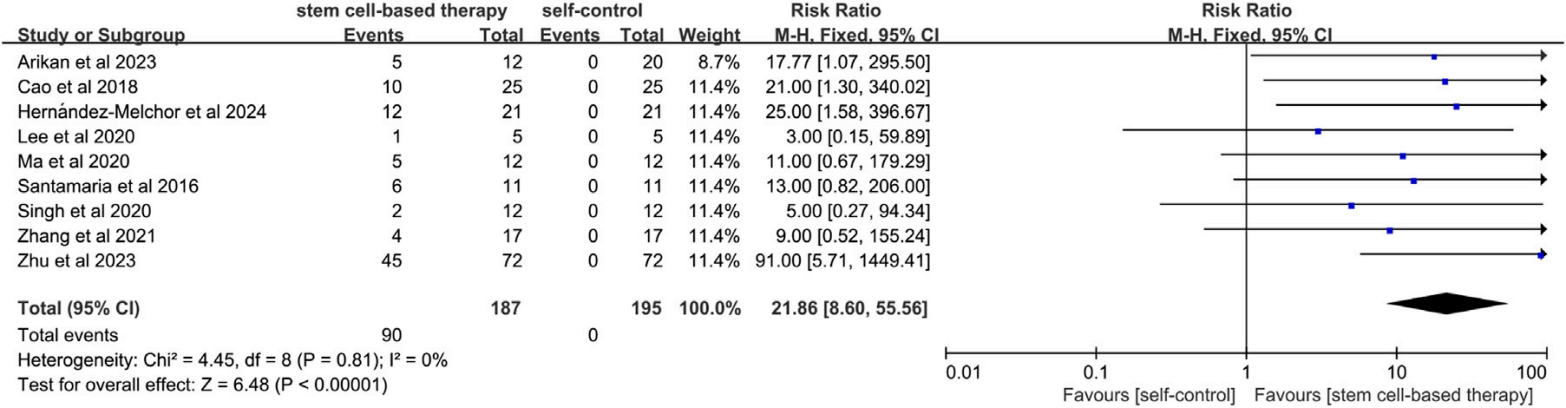
Forest plot of pregnancy outcome in stem cell therapy for IUA.

A total of nine studies mentioned clinical pregnancy rates. (Figure 3). Since the statistical heterogeneity among the studies was not significant (P = 0.81; I^2^ = 0%), the analysis of the pregnancy outcomes using a fixed-effects model showed that the clinical pregnancy rate in the stem cell treatment group was significantly better than that before treatment (RR = 21.86, 95% CI: [8.60, 55.56]). This suggests that stem cell therapy is effective in improving clinical pregnancy rates in patients with IUA. The results of sensitivity analysis showed that the results of meta-analysis were still statistically significant after excluding studies of high risk of bias (RR = 39.67, 95% CI [8.00, 196.68]) (Supplementary Figure S2).

#### 3.4.3 Live birth rate

**FIGURE 4:**
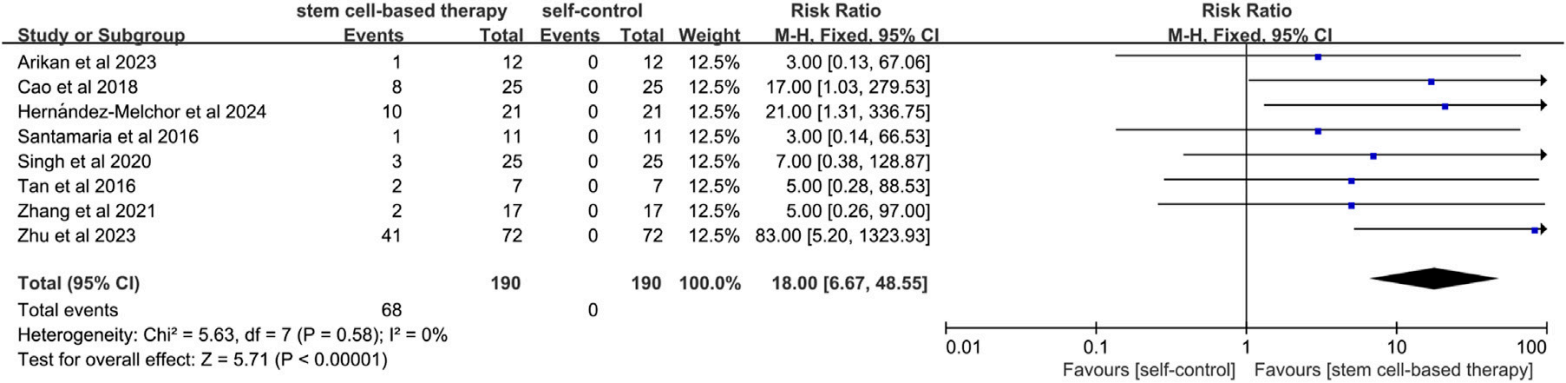
Forest plot of live birth rate in stem cell therapy for IUA.

A total of eight studies mentioned the comparison between stem cell treatment and self-control in terms of live birth rate. (Figure 4). Since the statistical heterogeneity among the studies was not significant (P = 0.58; I^2^ = 0%), a fixed-effects model was used, which showed that the live birth rate was significantly higher in the stem cell treatment group than in the control group (RR = 18.00, 95% CI: [6.67, 48.55]). This indicated that the effect of stem cell treatment on live birth rate was statistically significant. The results of sensitivity analysis showed that the results of meta-analysis were still statistically significant after excluding studies of high risk of bias (RR = 52.00, 95% CI [7.33, 368.83]) (Supplementary Figure S3).

#### 3.4.4 Miscarriage rate

**FIGURE 5:**
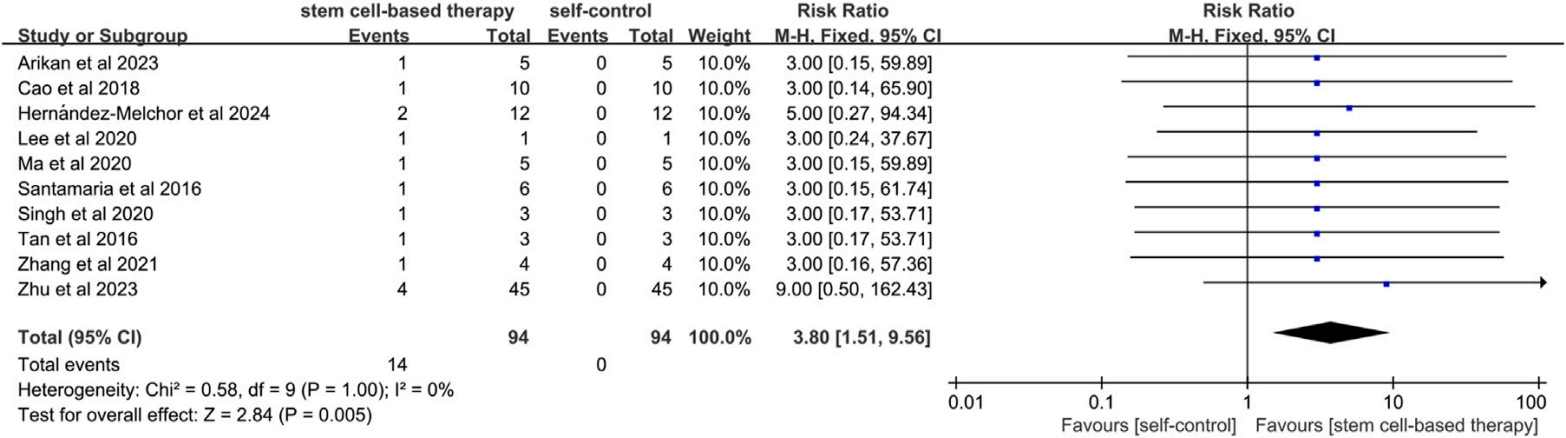
Forest plot of miscarriage rate in stem cell therapy for IUA.

A total of 10 studies referred to the comparison between stem cell treatment and self-control in terms of miscarriage rates. (Figure 5). Since the statistical heterogeneity between the studies was not significant (P = 1.00; I^2^ = 0%), a fixed-effects model was used and the results showed statistical significance (RR = 3.80, 95% CI: [1.51, 9.56]). The results of sensitivity analysis showed that there was no significant difference in miscarriage rate between the two groups after excluding studies of high risk of bias (RR = 5.67, 95% CI [1.10, 29.30]) (Supplementary Figure S4).

#### 3.4.5 Endometrial thickness

A total of 11 studies mentioned the effect of stem cell therapy in terms of endometrial thickness (Supplementary Figure S5). Due to the high statistical heterogeneity among the studies (P < 0.00001; I^2^ = 94%), a random-effects model was used and the results were statistically significant (MD = 2.28, 95% CI: [1.60, 2.96]). It demonstrated a significant advantage of stem cell therapy. The results of sensitivity analysis showed that the results of meta-analysis were still statistically significant after excluding studies of high risk of bias (MD = 2.13, 95% CI: [1.01, 3.26]) (Supplementary Figure S6). Due to the high statistical heterogeneity, we further explored the sources of heterogeneity. The results of meta-regression analysis showed that stem cell dose, rather than prior repair attempts, stem cell source, type of stem cells (autologous or allogeneic), injection methods and follow up is the source of heterogeneity (Supplementary Figures S7–12).

#### 3.4.6 Publication bias

For the indicators with the number of included studies greater than 10 were analyzed by funnel plots, the menstrual improvement funnel plot Supplementary Figure S13, and the miscarriage rate funnel plot Supplementary Figure S14 showed symmetrical distribution characteristics, and the p-value of the egger test was 0.34,0.94, respectively, which were greater than 0.05, indicating that the risk of publication bias was low. Endometrial thickness funnel plot Supplementary Figure S15 showed that the distribution of study sites was not symmetrical, and the egger test showed that the p-value was less than 0.05, supporting the possibility of publication bias. We therefore used the trim and fill method for endometrial thickness changes. The overall effect size corrected by trim and fill method was (MD = 1.0407, 95% CI: [0.3314; 1.7499], p = 0.0040). Although lower than the original effect size (MD = 2.28, 95% CI: [1.60, 2.96]), it suggests that the effect of stem cell treatment on endometrial thickness remained statistically significant even after accounting for publication bias and adjusting for it. Publication bias did not affect the stability of the results.

### 3.5 Subgroup analysis of the efficacy of different sources of stem cells

We performed subgroup analyses in multiple dimensions, including stem cell source, autologous allogeneic properties, injection method, and follow-up time, aiming to comprehensively assess the differences in the effectiveness of stem cell therapy in increasing endometrial thickness and to provide a detailed rationale for subsequent studies and clinical applications.

#### 3.5.1 Subgroup analysis of stem cell sources

In a subgroup analysis by stem cell source (Supplementary Figure S16), ADSCs involved two studies with results showing significant endometrial thickening (MD = 3.34, 95% CI: [2.55, 4.13]) and no heterogeneity (P = 0.86, I^2^ = 0%). The umbilical cord source group included 3 studies with results supporting effectiveness (MD = 1.39, 95% CI: [0.80, 1.98]) but with moderate heterogeneity (P = 0.08, I^2^ = 59%). The bone marrow source group included five studies with (MD = 2.27, 95% CI: [1.39, 3.16]) but significant heterogeneity (P < 0.00001, I^2^ = 94%). The group of menstrual blood sources contained only the Ma et al., 2020 single-center study (MD = 3.62, 95% CI: [3.03, 4.21]), which was not tested for heterogeneity due to insufficient sample size. In conclusion, adipose tissue- and blood-derived stem cells were highly effective in thickening the endometrium, whereas the heterogeneity of the umbilical cord-derived group, although effective, requires attention.

#### 3.5.2 Subgroup analysis of stem cell types (autologous/allogeneic)

Eight studies were included in the autologous stem cell group (Supplementary Figure S17). The random effects model results were (MD = 2.53, 95% CI: [1.67, 3.39]). The allogeneic stem cell group contained 3 studies with the result of (MD = 1.39, 95% CI: [0.80, 1.98]). That is, both autologous and allogeneic stem cells were effective in improving endometrial thickness, with the autologous group being more effective but further attention needs to be paid to the heterogeneous source.

#### 3.5.3 Subgroup analysis of injection modalities

The local injection group included 7 studies (Supplementary Figure S18), with a random effects model result of (MD = 2.70, 95% CI: [2.15, 3.25]) and moderate heterogeneity (P = 0.0005, I^2^ = 67%). The hysteroscopic adhesiolysis group contained 4 studies with a result of (MD = 1.46, 95% CI: [0.41, 2.51]) but with high heterogeneity (P < 0.00001, I^2^ = 97%).

#### 3.5.4 Short-term follow-up efficacy analysis

Of these, 10 studies were included in the 0–3 months analysis (Supplementary Figure S19). The results of which were (MD = 2.05, 95% CI: [1.12, 2.99]) and the test for heterogeneity showed a high degree of heterogeneity (P < 0.00001; I^2^ = 95%). A total of 3 studies were included in the 3–6 months subgroup analysis. The result was (MD = 1.99, 95% CI: [-0.70, 4.68]), and the test of heterogeneity showed a high degree of heterogeneity (P < 0.00001; I^2^ = 95%). In the analysis of >6 months, a total of 3 studies were included, resulting in (MD = 3.03, 95% CI: [1.83, 4.22]), and the results of the heterogeneity test showed a high degree of heterogeneity (P < 0.00001; I^2^ = 95%).

The overall results showed that the subgroup analyses of 0–3 months and >6 months showed a significant overall effect, with a statistically significant intervention effect at these times. In contrast, the overall effect of the subgroup analysis for 3–6 months was not significant.

### 3.6 Security evaluation

A total of two out of 12 studies (233 cases) reported the occurrence of adverse reactions. Of these, in the study by Singh et al. (2014), 3 out of 6 patients (50%) experienced adverse effects during treatment, with symptoms including loss of appetite, mild gastritis, abdominal cramps, and vomiting, which subsequently resolved on their own. In the study by Zhu et al. (2024a), out of 74 patients, nine patients (12.2%) reported postoperative lower abdominal pain, nine patients (12.2%) had placental adhesions that required manual peeling of the placenta. Two patients (2.7%) had abnormal white blood cell counts, five patients (6.8%) had abnormal neutrophil percentages, and three patients (4.1%) had abnormal neutrophil counts. It is worth noting that Zhu’s study (Zhu et al., 2024a) did not explicitly give the total number of adverse reactions, and there may be overlap in the data for different adverse reactions, i.e., the same person may have multiple adverse reactions. Taken together, none of the stem cell treatments caused serious complications. This suggests that stem cell therapy is safe to some extent.

## 4 Discussion

Twelve studies involving 233 patients were included in this study with the aim of evaluating the efficacy of stem cell therapy for IUA. Our systematic analysis of this study showed that stem cell therapy demonstrated significant clinical value in improving the reproductive prognosis of patients with IUA. Specific results showed that stem cell therapy had significant advantages in improving menstruation, improving pregnancy outcome, increasing live birth rate, decreasing miscarriage rate and increasing endometrial thickness. Meanwhile, this study separated the studies according to stem cell source, autologous allogeneic, injection method, and follow-up time in order to achieve a more precise clinical efficacy analysis, and the results of the subgroup analysis were generally statistically significant.

Due to limited available data, we did not conduct a meta-analysis on IUA score changes. A total of 3 studies described changes in IUA scores, all using the American Fertility Society (AFS) IUA scoring system. Among them, Cao’s study (Cao et al., 2018b) showed that the mean IUA score of 26 patients was 9.12 ± 1.51 before treatment. 3 months after treatment as the mean score decreased to 5.52 ± 3.22 (p < 0.01).In Santamaria’s study (Santamaria et al., 2016) out of 11 patients, 3 had grade III, 4 had grade II (combined with endometrial atrophy), and the rest had grade I. In the study, 3 patients had grade II, and the rest had grade II. Three months after treatment, all four grade III patients improved to grade I. One grade II patient had normalization of the uterine cavity and one improved to grade I. In the study of Santamaria at, out of 11 patients, 1 had endometrial atrophy and the rest had grade I. In Zhu’s study (Zhu et al., 2024a), the median reduction in AFS scores before and after stem cell therapy was −7 (−8 to −5) in 74 patients. The results of these studies suggest that IUA scores were reduced to varying degrees after stem cell therapy, and the improvement was particularly significant in patients with moderate to severe IUA. Despite the limited data, the available evidence supports the effectiveness of stem cell therapy in reducing IUA scores.

Our study is consistent with previous Meta-analyses (Zhao et al., 2020; Chen et al., 2022) in terms of core findings, both confirming that stem cell therapy significantly improves menstrual status, pregnancy outcome, and endometrial thickness in patients with IUA, and no serious adverse effects were reported; however, conditions such as placenta adherence, as seen in the (Zhu et al., 2024a) study, need to be emphasized. Twelve studies (233 cases) were included in this paper, which significantly enlarged the sample size from previous analyses (8–10, 84–116 cases). For the first time, the live birth rate and miscarriage rate were systematically assessed, confirming the value of stem cell therapy in improving the long-term prognosis of reproduction and filling a gap in the understanding of long-term outcomes of reproduction. The efficacy advantages of adipose (MD = 3.34, 95% CI: [2.55–4.13]) and menstrual blood-derived (MD = 3.62, 95% CI: [3.03–4.21]) stem cells were also revealed.

The dominant treatment for Asherman syndrome in the past has been hysteroscopic adhesiolysis, as it can help to separate uterine fissures and thus enlarge the uterine cavity for conception. However, there is a risk of inducing residual endometrial damage and accelerating scar formation during the procedure (Yu et al., 2008; Jansen et al., 2000) and it has a limiting role in treating certain unsuccessful reproductive disorders (Deans et al., 2018). Therefore, there is an urgent need to establish a new clinical treatment to repair the damaged uterus. In recent years, stem cells have emerged as a new treatment for IUA due to their ability to differentiate and promote endometrial regeneration (Shao et al., 2019). Stem cells have multidirectional differentiation potential and can differentiate into endometrial cells to directly repair damaged endometrial tissues, and their immunomodulatory effects can reduce inflammation and promote endometrial regeneration and repair; Stem cells possess multidirectional differentiation potential, enabling direct differentiation into endometrial cells to repair damaged tissues. Additionally, their immunomodulatory effects reduce inflammation and promote endometrial regeneration (Gharibeh et al., 2022; Tan et al., 2016). Although more clinical trials are needed to prove the efficacy of stem cell therapy, it also brings hope for improving the reproductive prognosis of patients with IUA.

The results of our meta-analysis showed that stem cell therapy significantly improved patients' menstrual situation. In the included clinical studies, patients with IUA, all of whom had menstrual atresia or insufficient menstrual flow, showed significant improvement in most of them after stem cell therapy compared with their own or blank control groups. A phase I clinical trial of 12 patients with severe IUA showed that all patients experienced prolonged and increased menstrual flow in the first menstrual cycle after *in utero* transplantation of UC-MSCs, and menstruation was restored in patients with amenorrhea (Ma et al., 2020). It has been suggested in the literature that MSCs-derived exosomes promote angiogenesis and ultimately increase endometrial microvessel density by promoting the expression of endothelial cell growth factors (e.g., VEGF, FGF, etc.) through angiogenesis-related miRNAs (e.g., miR-29b) they carry (Li et al., 2016).

Our analysis demonstrates that stem cell therapy is effective in improving pregnancy outcomes in patients with IUA and can greatly increase the live birth rate. A clinical trial on IUA was published, in which 72 patients with IUA were treated with autologous BMSCs for 2 years, 62.5% of the participants sustained a pregnancy with a live birth rate of 56.9% (Zhu et al., 2024b).Our analysis demonstrates that stem cell therapy is effective in improving pregnancy outcomes in patients with IUA and can greatly increase the live birth rate and decrease the miscarriage rate. A clinical trial on IUA was published, in which 72 patients with IUA were treated with autologous BMSCs for 2 years, 62.5% of the participants sustained a pregnancy with a live birth rate of 56.9% (Cao et al., 2018a). This significant improvement in reproductive prognosis may be due to the fact that in the IUA model, hUC-MSCs transplanted with hUC-MSCs can exert diverse differentiation potentials to promote endometrial repair and increase angiogenesis, as well as secretion of VEGF and bFGF to improve the local microenvironment and endometrial receptivity, which ultimately improves the infertility and miscarriage of patients.

The main pathological mechanism of IUA is the damage to the endometrial basal layer leading to dysfunction of the functional layer repair, which in turn triggers fibrous connective tissue proliferation and endometrial fibrosis, ultimately leading to a decrease in endometrial thickness (Liu and Wang, 2022). Our meta-analysis showed that endometrial thickness in patients with IUA increased significantly after stem cell treatment. A clinical trial on IUA, in which 12 patients with IUA were treated with UC-MSCs, showed a significant increase in endometrial thickness from 3.9 ± 0.9 mm to 7.5 ± 0.6 mm (P < 0.001) (Ma et al., 2020). MSCs can be successfully isolated from a wide variety of adult tissues including bone marrow, adipose tissues, umbilical cords, and menstrual blood. And MSCs from different sources may have differences in surface marker expression, differentiation potential and paracrine function, which may affect their effectiveness in IUA treatment (Li and Hu, 2023). Therefore, we conducted subgroup analysis according to the different sources of stem cells, and the results showed that all four sources of stem cells could increase endometrial thickness, and the effect of adipose tissue- and menstrual blood-derived stem cells was particularly significant (Supplementary Figure S11). MSCs from adipose tissue and menstrual blood sources are relatively non-invasive and have a strong value-added capacity (Cao et al., 2021; Klingemann et al., 2008), in which adipose-derived P1 generation cells take 15–22 days to grow to their full size, and their proliferation capacity is close to that of amniotic fluid MSCs, and their immune-modulating capacity is significantly better than that of BMSCs. Therefore, in the future, we can prioritize adipose tissue- and menstrual blood-derived MSCs, which have a better therapeutic efficacy and are more non-invasive, in the treatment of patients with IUA. MSCs. Exosomes derived from placental mesenchymal stem cells (PMSCs) can restore uterine function and improve fertility in injured animals by promoting cell proliferation, increasing endometrial thickness and reversing fibrosis, according to a recent study (Liu et al., 2024).

Our result showed that both autologous and allogeneic stem cells had significant efficacy in increasing endometrial thickness (Supplementary Figure S12), a result highly consistent with previous studies. Autologous stem cells have been shown to be effective in repairing the endometrium in several clinical studies due to their low immunogenicity, lack of ethical controversy, and the fact that they can be obtained directly from the patient’s bone marrow or menstrual blood. For example, in a clinical trial at Nanjing Gulou Hospital, autologous bone marrow stem cells combined with collagen scaffold transplantation led to successful conception in 13 patients with severe IUA, with a significant increase in endometrial thickness (from 4.2 mm to 7.5 mm on average) after the procedure (Cao et al., 2018a; Gharibeh et al., 2022). While allogeneic stem cells (e.g., UC-MSCs) have the risk of immune rejection, the growth factors secreted by them, such as VEGF and HGF, can promote neovascularization and improve the local microenvironment through paracrine effects. A phase I clinical trial of 26 patients showed that 3 months after transplantation of UC-MSCs, the endometrial thickness of patients increased by 2.1 mm (p < 0.001), and no serious adverse reactions occurred. Subgroup analysis showed that the efficacy of the local injection group was superior to that of the hysteroscopic adhesiolysis group (Supplementary Figure S13). This difference may stem from the effect of the two delivery modes on stem cell survival and function. Local injection can avoid the cell loss caused by uterine fluid flushing by the high concentration of stem cells acting directly on the injured area. Single-cell transcriptome studies have shown that perivascular cell senescence and basement membrane thickening are significant in thin endometrium, and local injection can inhibit fibrosis through the paracrine SEMA3 signaling pathway while activating the EGF pathway to promote epithelial proliferation (Lv et al., 2022). In contrast, although hysteroscopic adhesiolysis can evenly distribute stem cells, it may reduce cell activity due to mechanical stimulation or changes in the immune microenvironment. For example, an animal study found that transient elevation of local inflammatory factors (e.g., TNF-α) after hysteroscopic manipulation may inhibit stem cell survival (Sapozhak et al., 2020). There is also an interesting study that mentions that *in vivo*, transplantation of endometrial stromal cells (EnSCs) promotes structural and functional recovery of the endometrium and improves pregnancy rates, and that EnSCs and MSCs have similar characteristics, so an analogy can be made (Zhou et al., 2024).

Both the 0–3 months and >6 months subgroups showed significant efficacy (Supplementary Figure S14), suggesting a biphasic mode of action of stem cell therapy. The short-term effect (0–3 months) may stem from the rapid paracrine effects of stem cells. For example, a clinical study showed that a 1.8-fold increase in VEGF levels (p = 0.003) could be detected within 6 days after stem cell transplantation, promoting neovascularization (Sapozhak et al., 2020). Long-term effects (>6 months) were then associated with stem cell differentiation and tissue remodeling. Long-term follow-up data from Nanjing Gulou Hospital showed that patients' endometrial thickness remained stable (mean 7.2 mm) 12–24 months after transplantation, with neoplastic glands and vascular networks visible on histology (Cao et al., 2018a). Single-cell sequencing revealed that stem cells could sustainably activate the endometrial regeneration program by upregulating LCN2+/SAA1+ epithelial subpopulation markers. In addition, reprogramming of the immune microenvironment (e.g., increased proportion of regulatory T cells) may suppress chronic inflammation and maintain long-term repair (Ren et al., 2022).

This study showed that stem cell therapy significantly improved menstrual recovery, pregnancy success, live birth and endometrial thickness in patients with IUA. However, the ROBINS-I tool evaluation showed that 75% of the included original studies had high-risk bias. This poses a serious challenge to the internal validity and reliability of the results. A high proportion of the risk of bias means that there are significant limitations in the existing evidence base, which may overestimate or underestimate the estimation of the real effect. However, the results of sensitivity analysis showed that after excluding high-risk bias studies, the main research results did not reverse the direction and were still statistically significant, indicating the robustness of the current research results. Although the results suggest potential association signals, any causal inference should be extremely cautious due to the high risk of bias of the evidence. In the future, it is urgent to design rigorous and well-controlled prospective cohort studies or randomized trials to provide more reliable evidence. Although this analysis confirmed that stem cell therapy had a significant effect in the treatment of IUA, data on adverse reactions were still seriously lacking. Only two studies reported adverse reactions, and no adverse reactions were graded. Due to data heterogeneity, it is difficult to quantitatively synthesize and estimate the incidence of adverse reactions at all levels, which reduces the level of evidence for this result. In the future, the quantitative data pool of adverse reactions of stem cell therapy for IUA needs to be expanded. In addition, the long-term safety of stem cell therapy (especially the risk assessment of tumorigenicity after allogeneic transplantation) and the optimal treatment parameters (such as cell dose, transplantation interval) still need to be further verified. Future studies could focus on integrating single-cell multi-omics technology to screen stem cell subpopulations with immunomodulatory properties (e.g., PD-L1 high-expression population), and optimizing the cell delivery system by combining with smart biomaterials (e.g., temperature-sensitive hydrogel), so as to enhance the efficacy and reduce the potential risks, and to provide a more precise strategy for clinical translation.

There are some limitations in this study. First of all, although a comprehensive literature search has been conducted, some unpublished or grey literature may still be missed, resulting in potential publication bias, which may affect the accurate estimation of the effect value of meta-analysis. Secondly, the quality of the included studies is uneven. Some studies have shortcomings in randomization, blind implementation and sample size. More than half of the literature has a high risk of bias. Although the sensitivity analysis confirms the robustness of the results, the conclusions of this study still need to be carefully explained. Third, the serious lack of safety data limits the quantitative synthesis of meta-analysis and reduces the evidence level of the results. It is urgent to expand the quantitative data pool of related adverse reactions. Fourthly, the definitions and measurement methods of outcome indicators in different studies are not completely consistent, which increases the difficulty of data merging and analysis, and introduces potential clinical heterogeneity, thus affecting the accurate interpretation of the results. Finally, since most of the included studies were short-term follow-up, there was a lack of evaluation of the long-term efficacy and safety of stem cell therapy, and the long-term effects of treatment could not be determined.

## 5 Conclusion

This study showed that stem cell therapy may improve menstrual recovery, pregnancy success, live birth and endometrial thickness in patients with IUA. Among them, adipose tissue and menstrual blood-derived stem cells showed superior endometrial repair Although autologous stem cells are more effective than allogeneic, the risk of placental adhesions after allogeneic transplantation should be cautioned. Current evidence supports the preference for local injection of autologous adipose/menstrual blood-derived stem cells as a treatment option. Due to the lack of data, the safety of stem cell therapy for IUA needs to be further verified.

However, existing studies have limitations such as predominantly short-term follow-up (≤6 months), small sample sizes and high heterogeneity, especially lacking comparative data between different stem cell types (e.g., ADSCs vs. UC-MSCs). Future multicenter RCTs focusing on evaluating long-term reproductive outcomes (e.g., secondary adhesion rate), optimizing cell delivery systems (e.g., bioscaffold coapplication), and establishing efficacy prediction models based on single-cell sequencing are needed to achieve precise and individualized treatment.

## Data Availability

The original contributions presented in the study are included in the article/Supplementary Material, further inquiries can be directed to the corresponding author.
