# Supplementary material for: Meta-analysis of mesenchymal stem cell therapy for intrauterine adhesions: a comprehensive consideration of efficacy and safety
Source: Front Bioeng Biotechnol. 2025 Aug 13;13:1619778. doi: 10.3389/fbioe.2025.1619778 (PMC12381504; doi:10.3389/fbioe.2025.1619778)
Supplement: Supplementary file 1 [file Supplementaryfile1.docx]

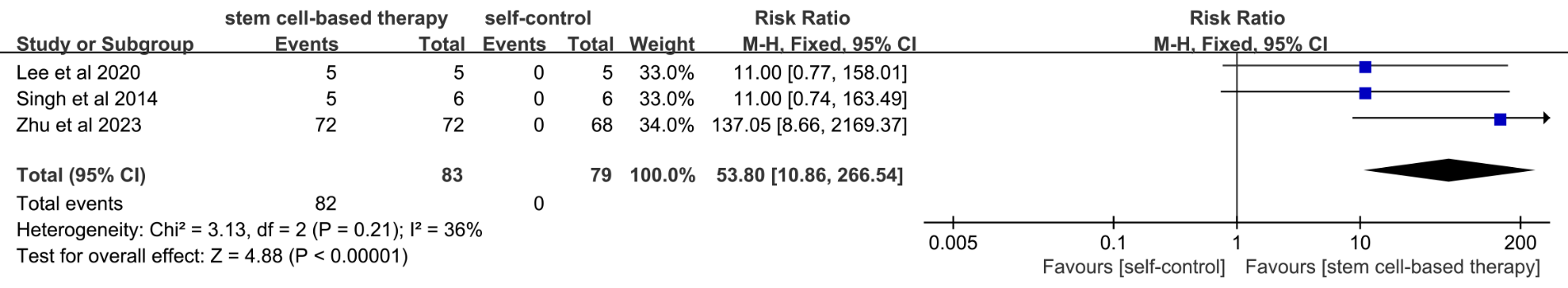


Supplementary Figure 1. Forest plot of menstrual improvement in stem cell therapy for IUA after excluding studies of high risk of bias.


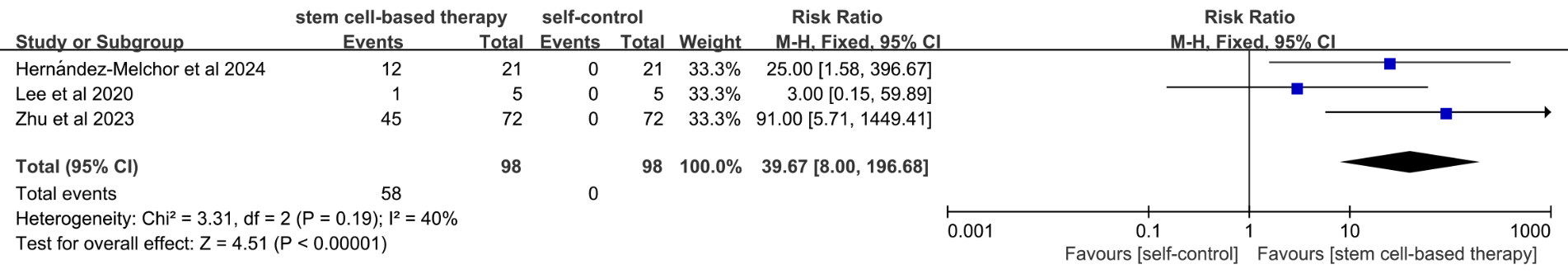


Supplementary Figure 2. Forest plot of clinical pregnancy rate in stem cell therapy for IUA after excluding studies of high risk of bias.


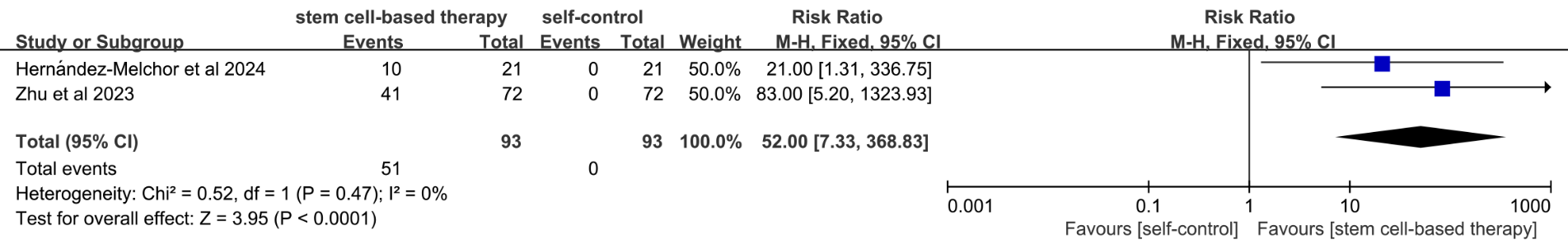


Supplementary Figure 3. Forest plot of live birth rate in stem cell therapy for IUA after excluding studies of high risk of bias.


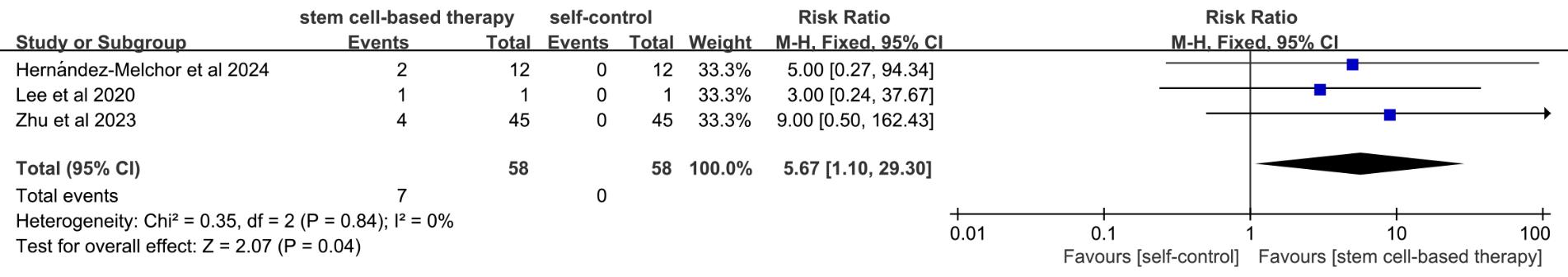


Supplementary Figure 4. Forest plot of miscarriage rate in stem cell therapy for IUA after excluding studies of high risk of bias.


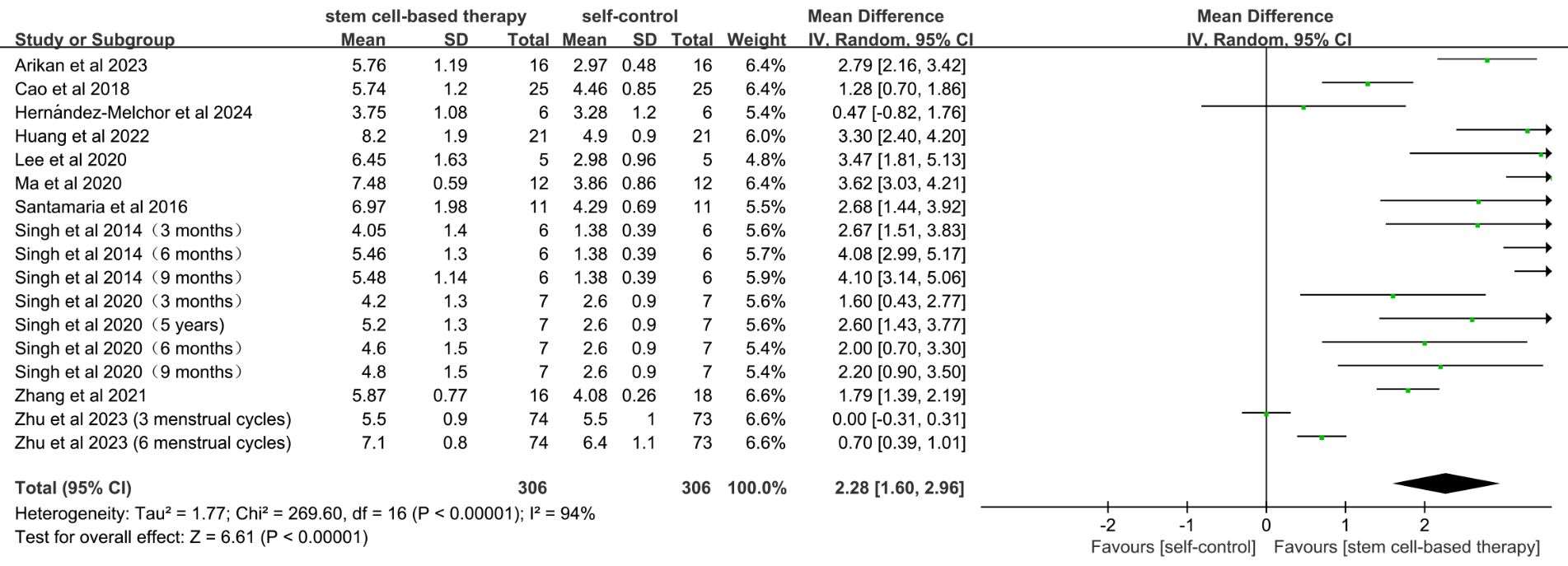


Supplementary Figure 5. Forest plot of endometrial thickness in stem cell therapy for IUA.


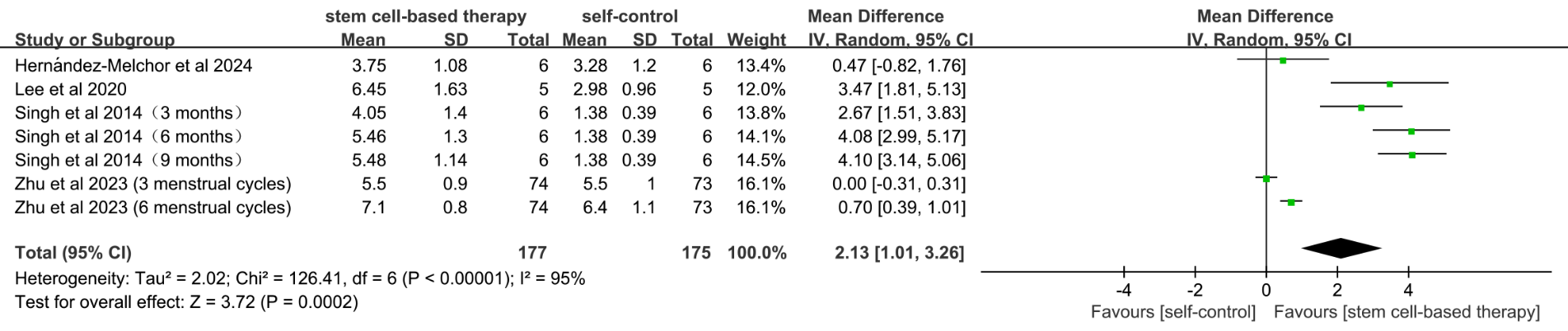


Supplementary Figure 6. Forest plot of endometrial thickness in stem cell therapy for IUA after excluding studies of high risk of bias.


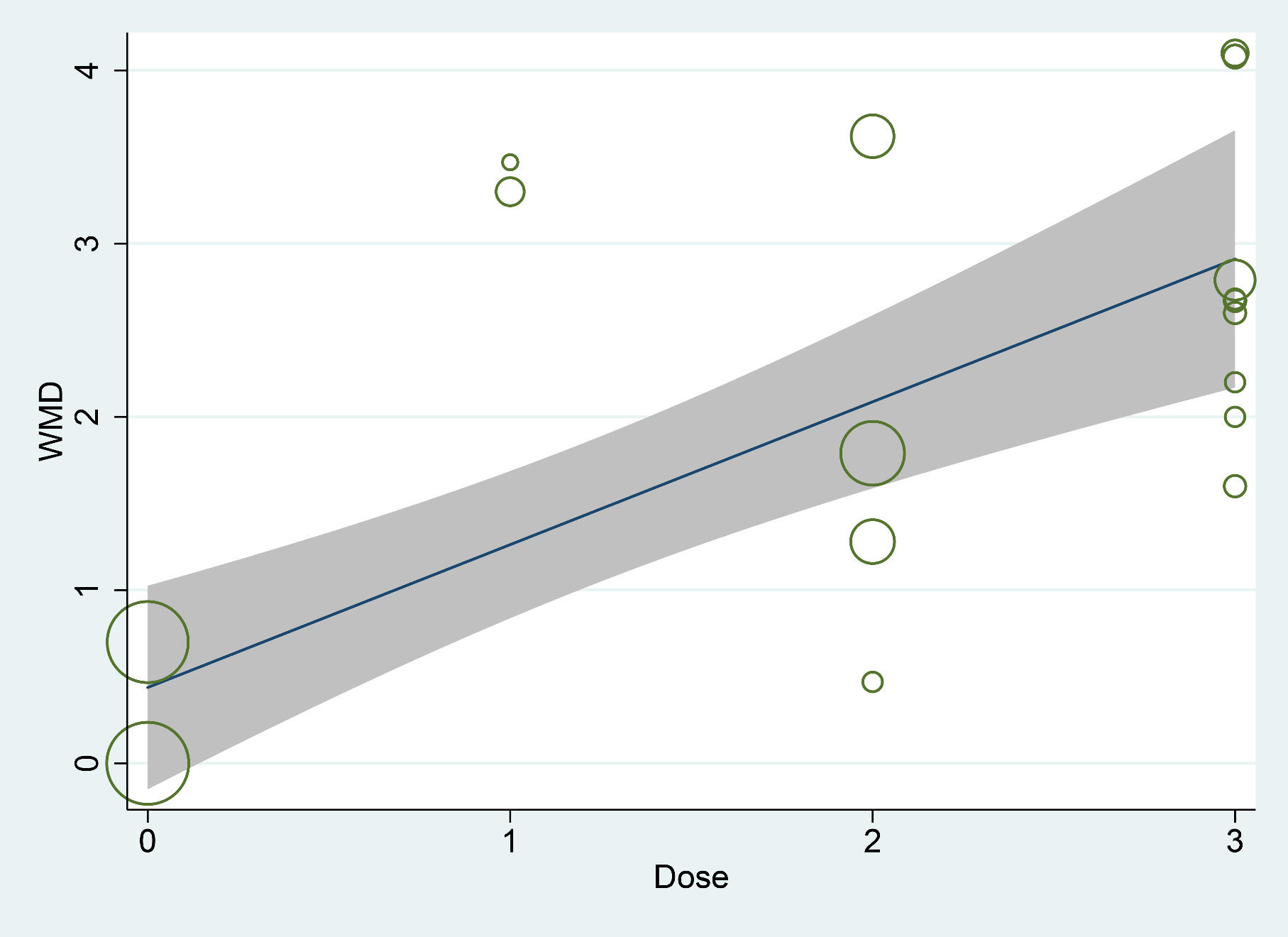


Supplementary Figure 7. Meta regression analysis: the effect of stem cell dose on endometrial thickness.


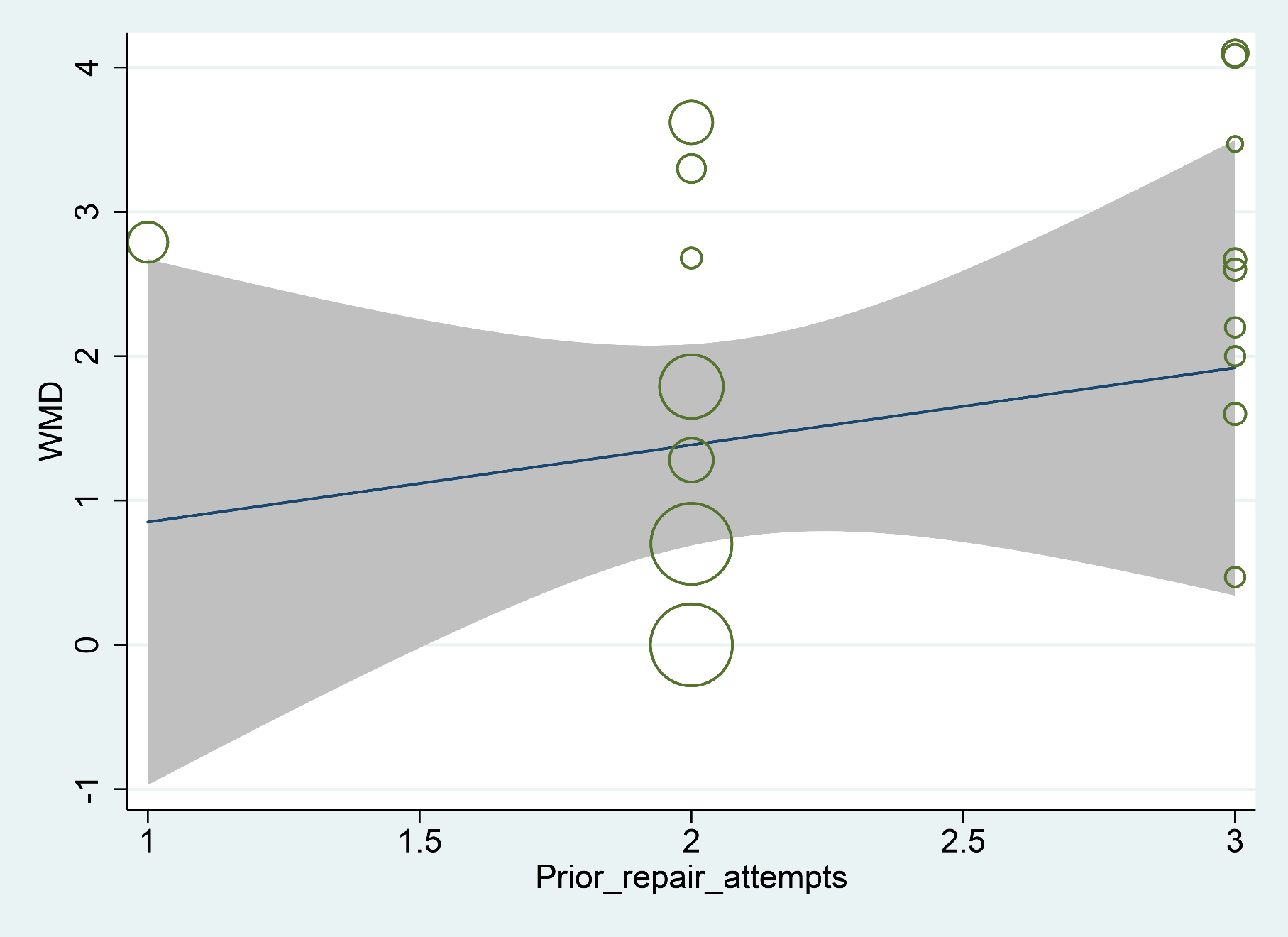


Supplementary Figure 8. Meta regression analysis: the effect of prior repair attempts on endometrial thickness.


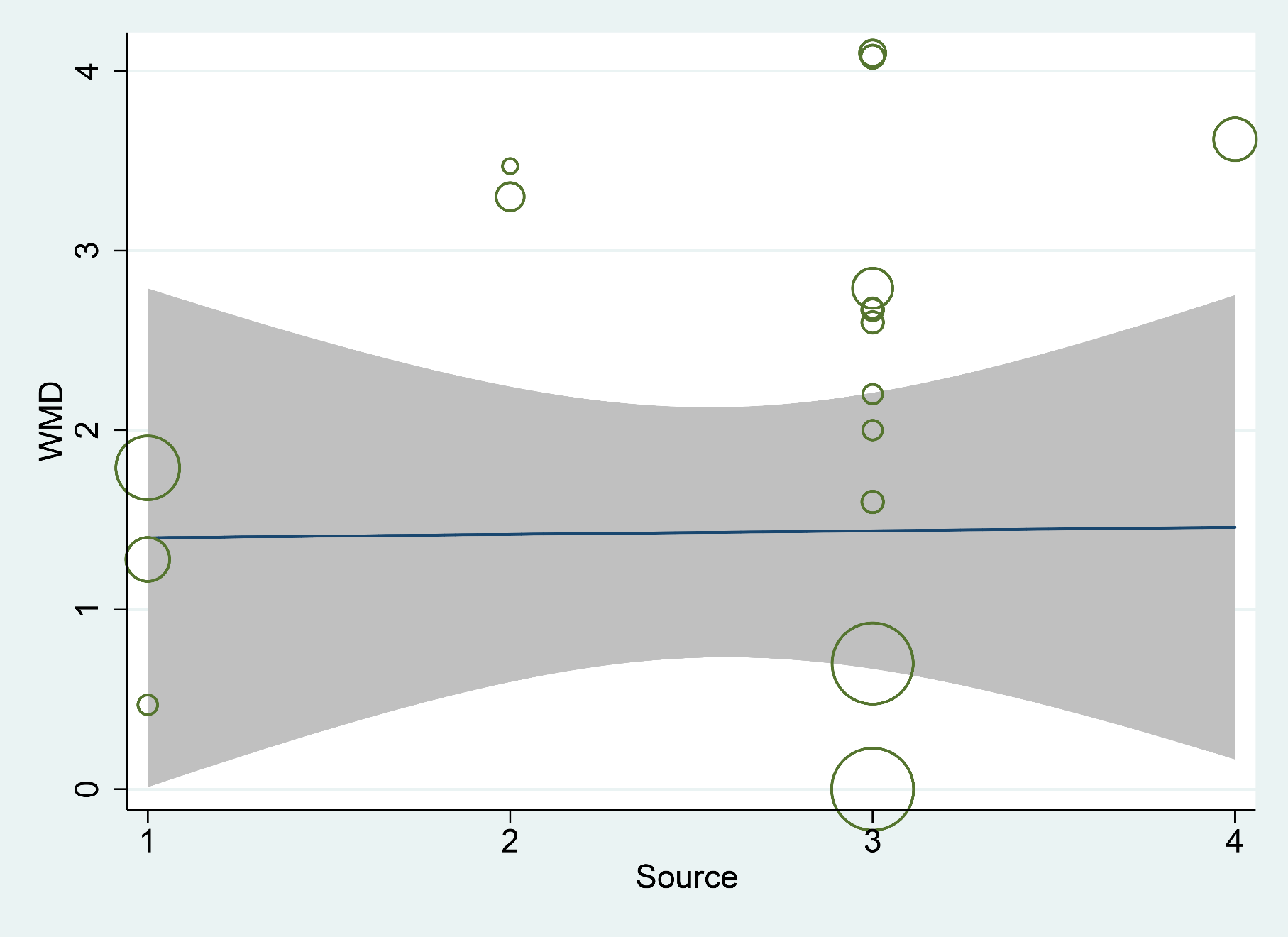


Supplementary Figure 9. Meta regression analysis: the effect of stem cell source on endometrial thickness.


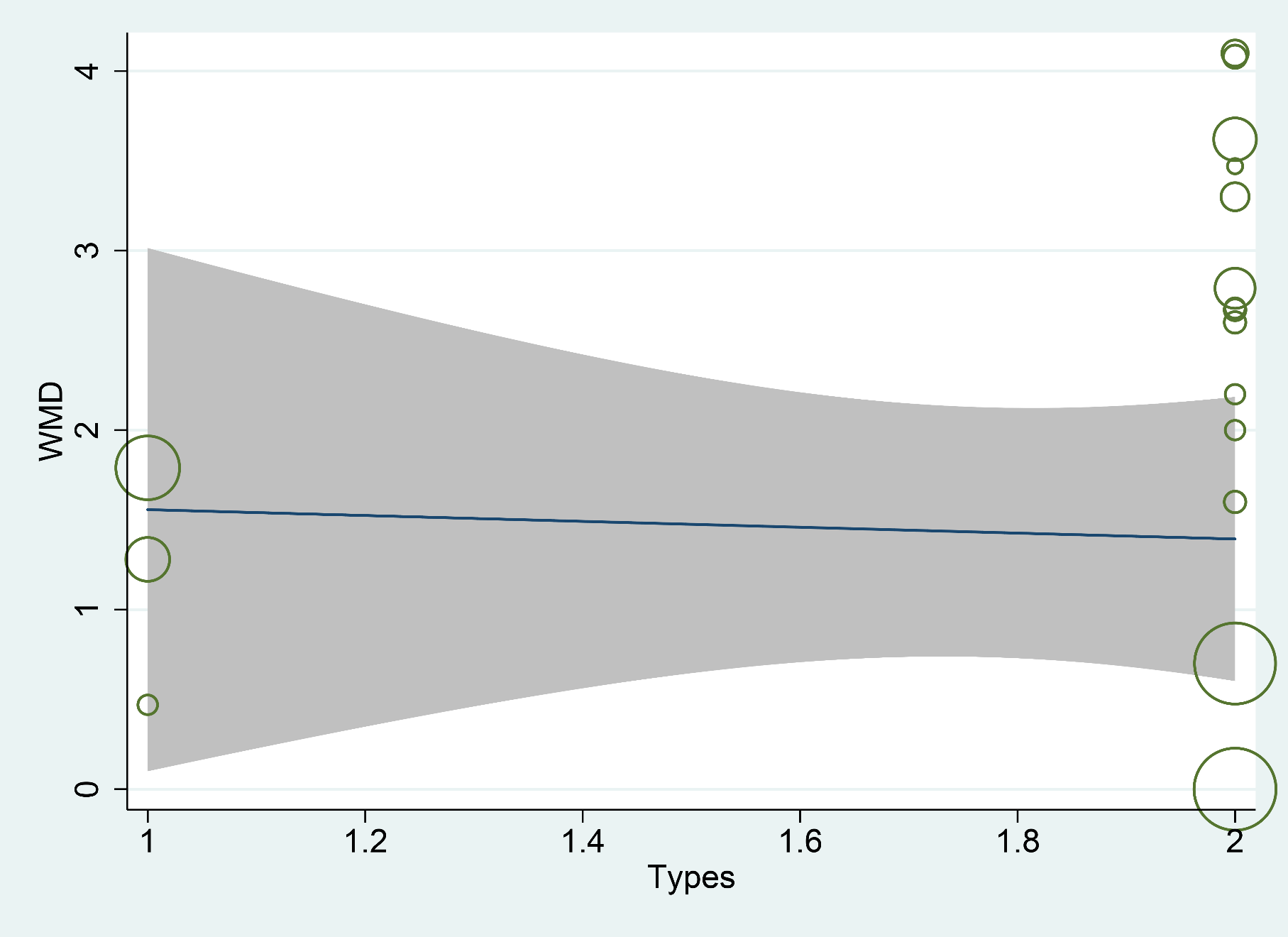


Supplementary Figure 10. Meta regression analysis: the effect of stem cells types (autologous or allogeneic) on endometrial thickness.


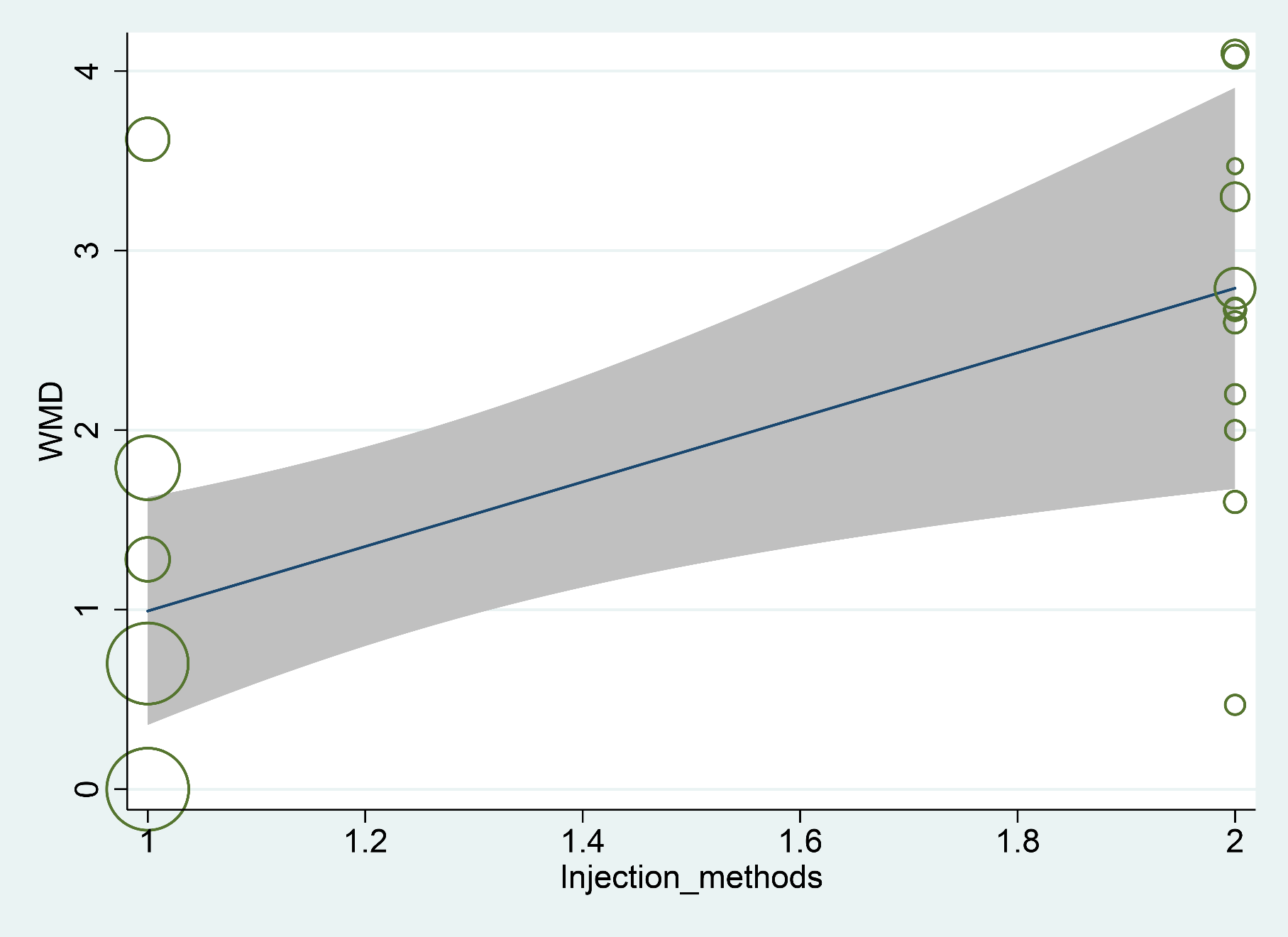


Supplementary Figure 11. Meta regression analysis: the effect of injection methods of stem cells on endometrial thickness.


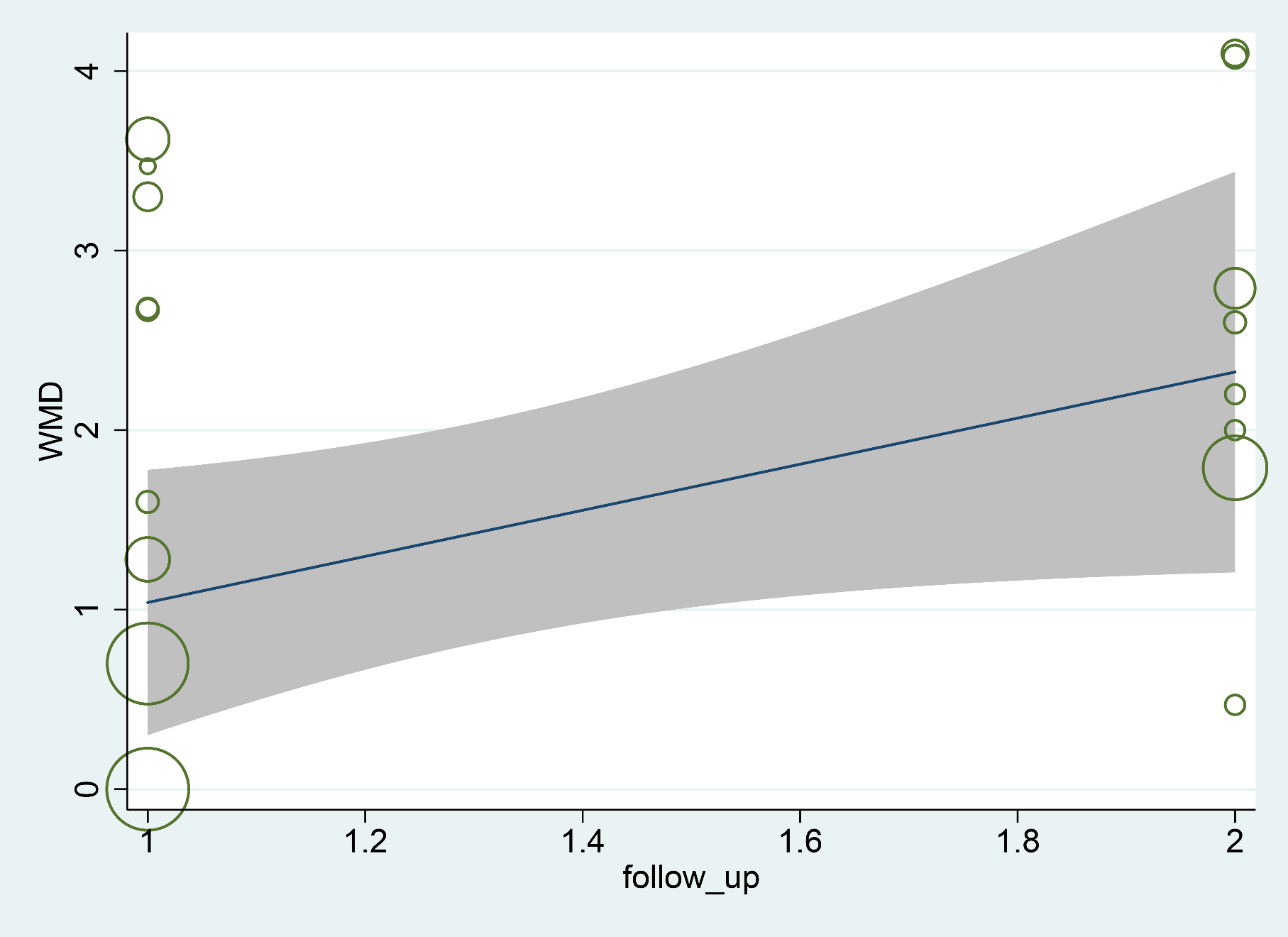


Supplementary Figure 12. Meta regression analysis: the effect of follow up on endometrial thickness.


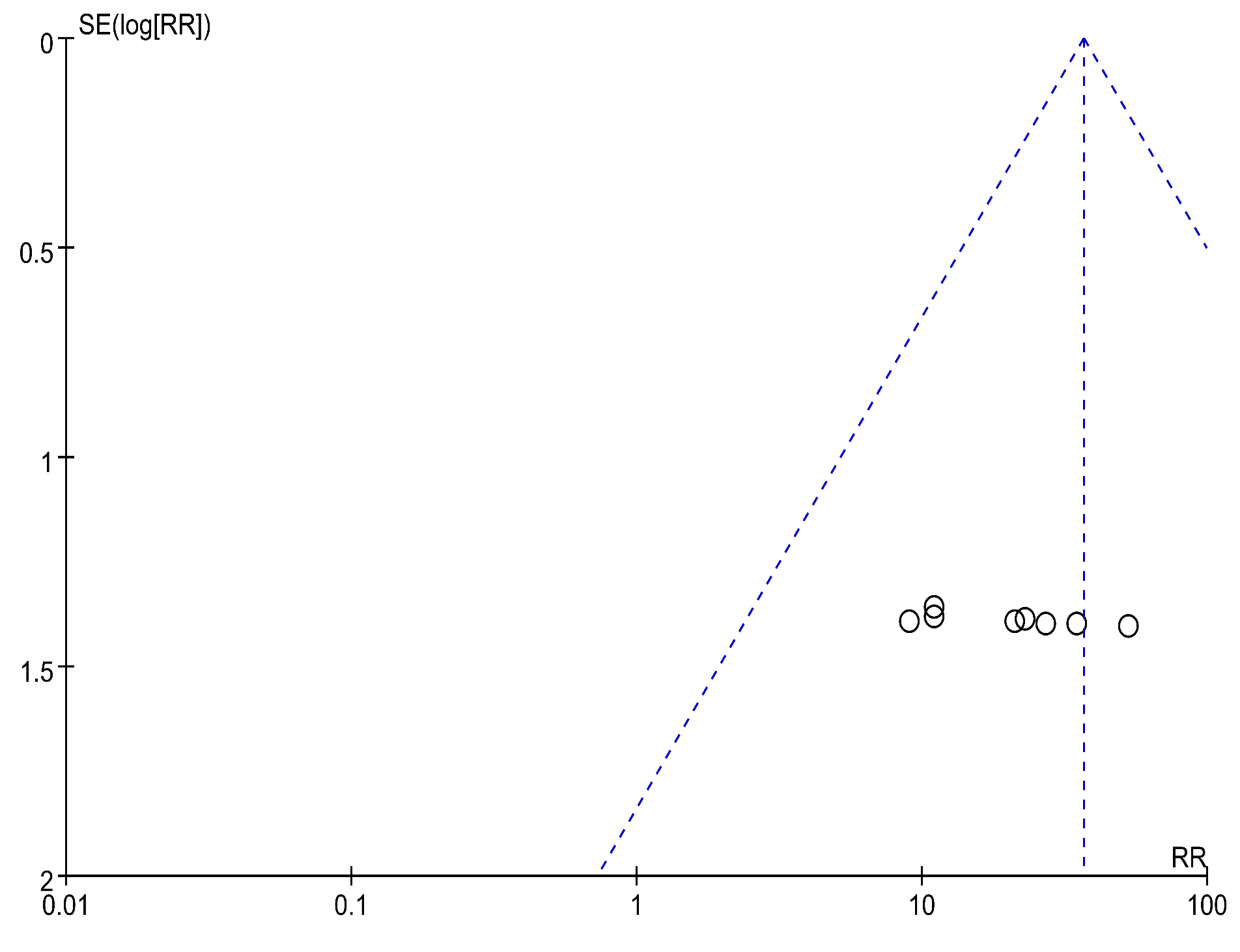


Supplementary Figure 13. Funnel plot of menstrual Improvement.


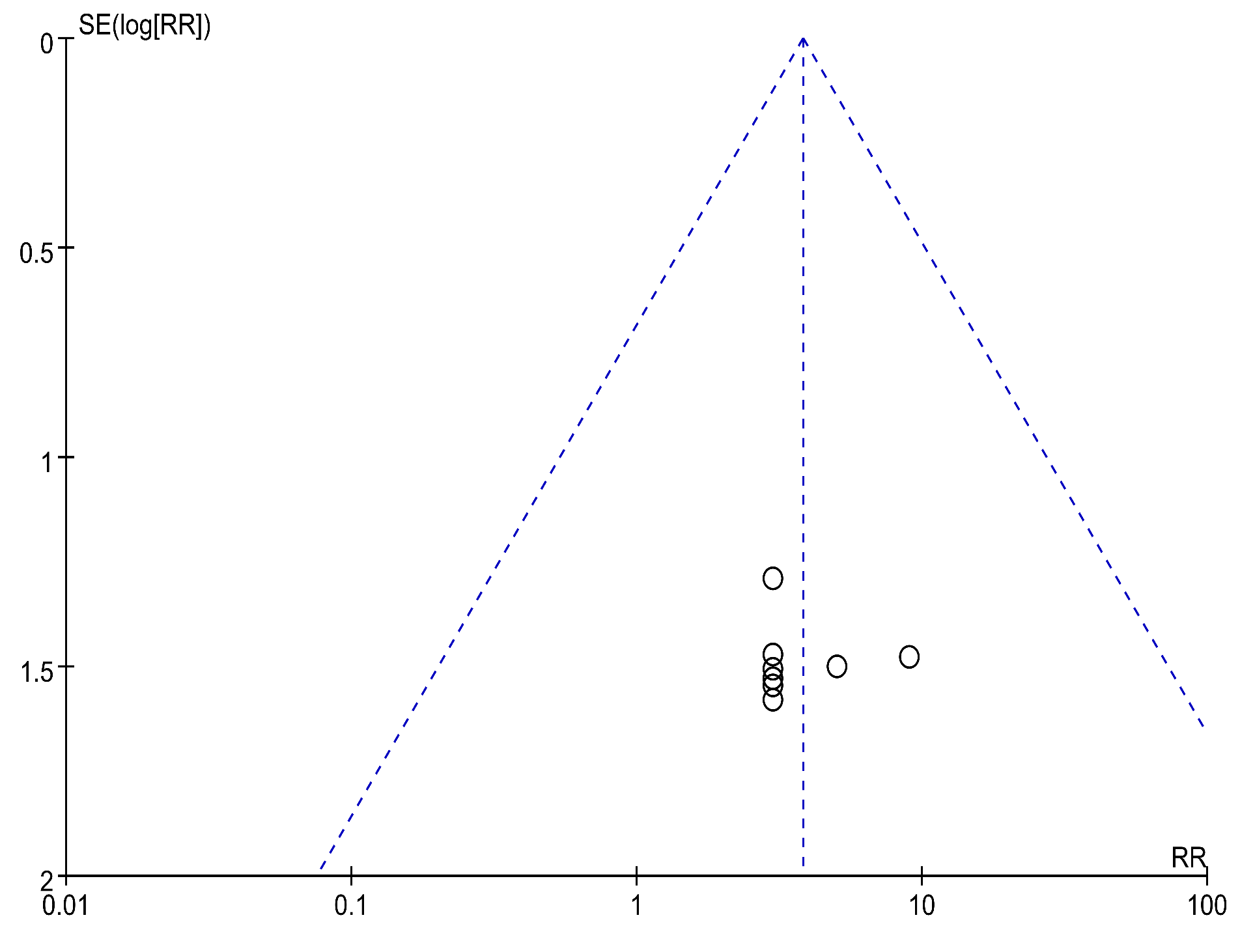


Supplementary Figure 14. Funnel plot of abortion rate.


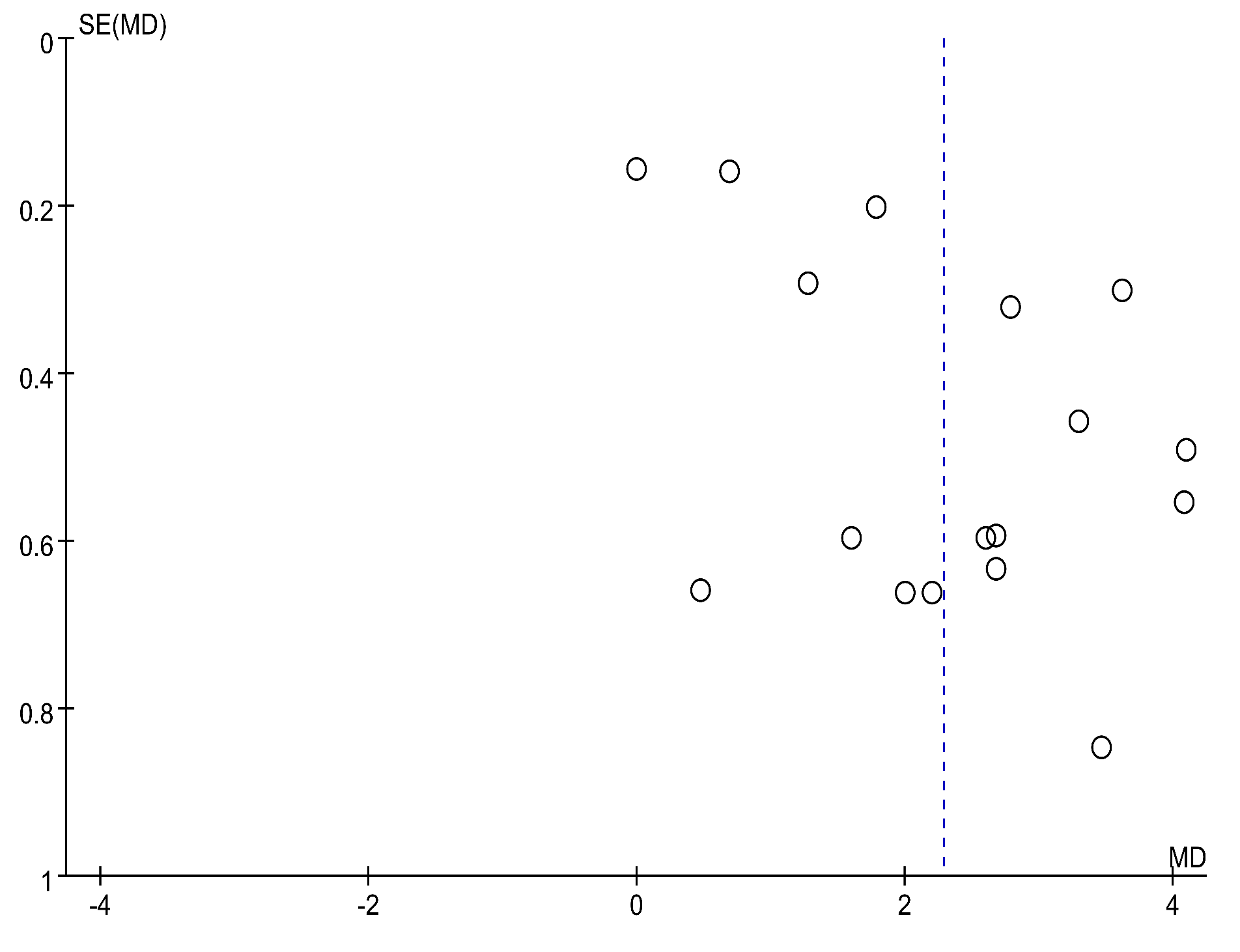


Supplementary Figure 15. Funnel plot of endometrial thickness.


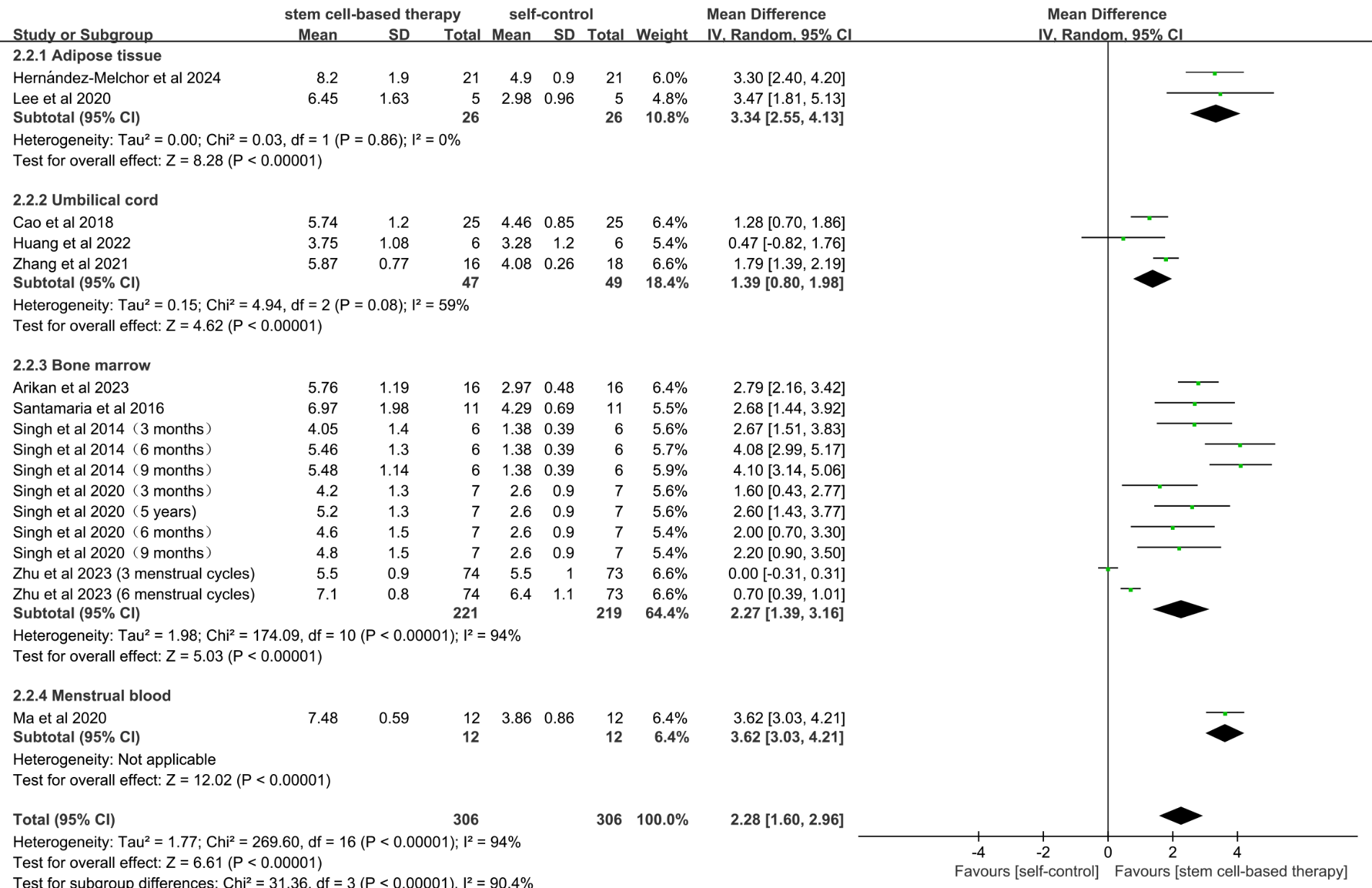


Supplementary Figure 16. Subgroup analysis of endometrial thickness improvement with different stem cell sources.


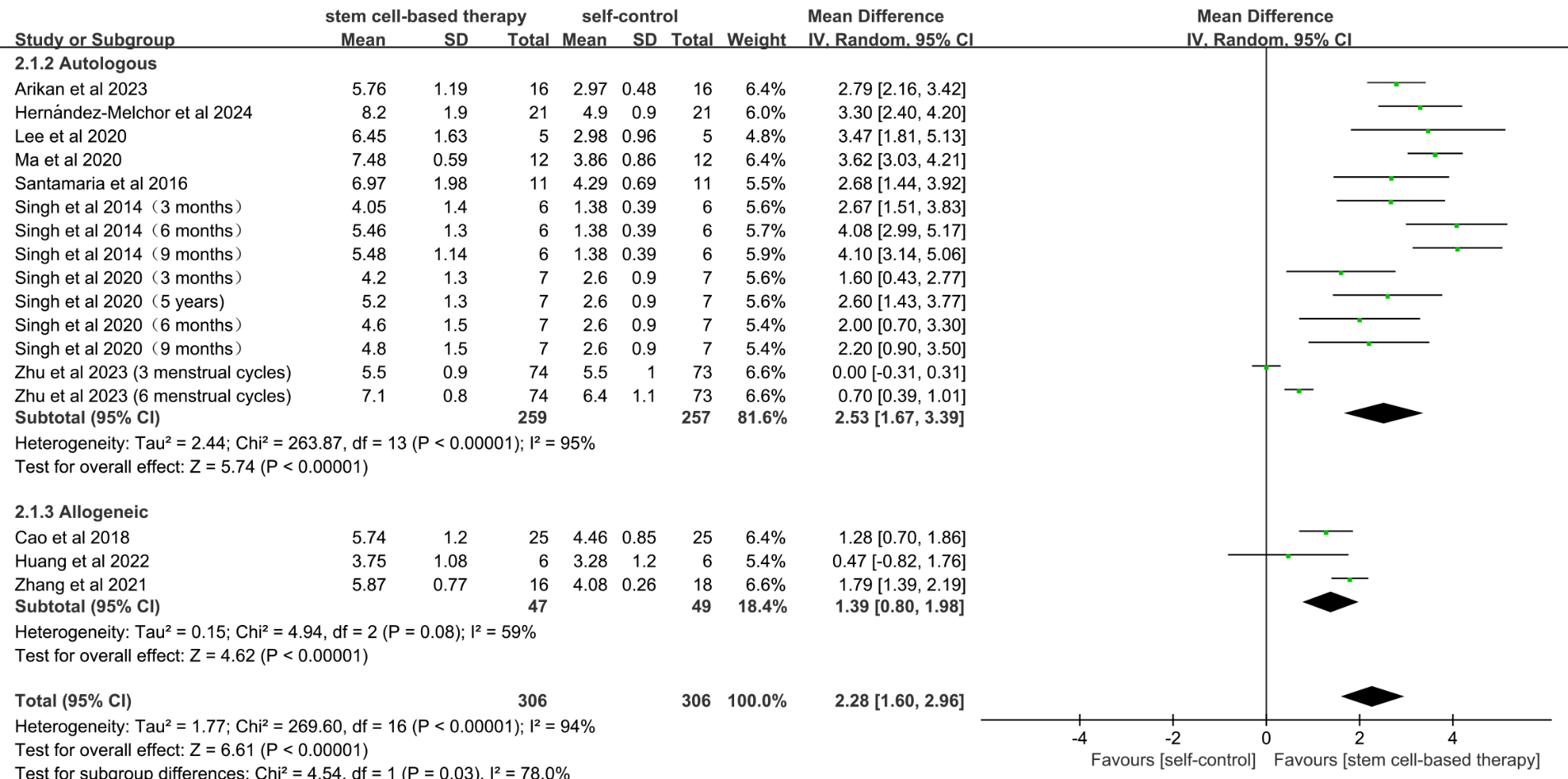


Supplementary Figure 17. Subgroup analysis of endometrial thickness improvement with autologous stem cell and allogeneic stem cell.


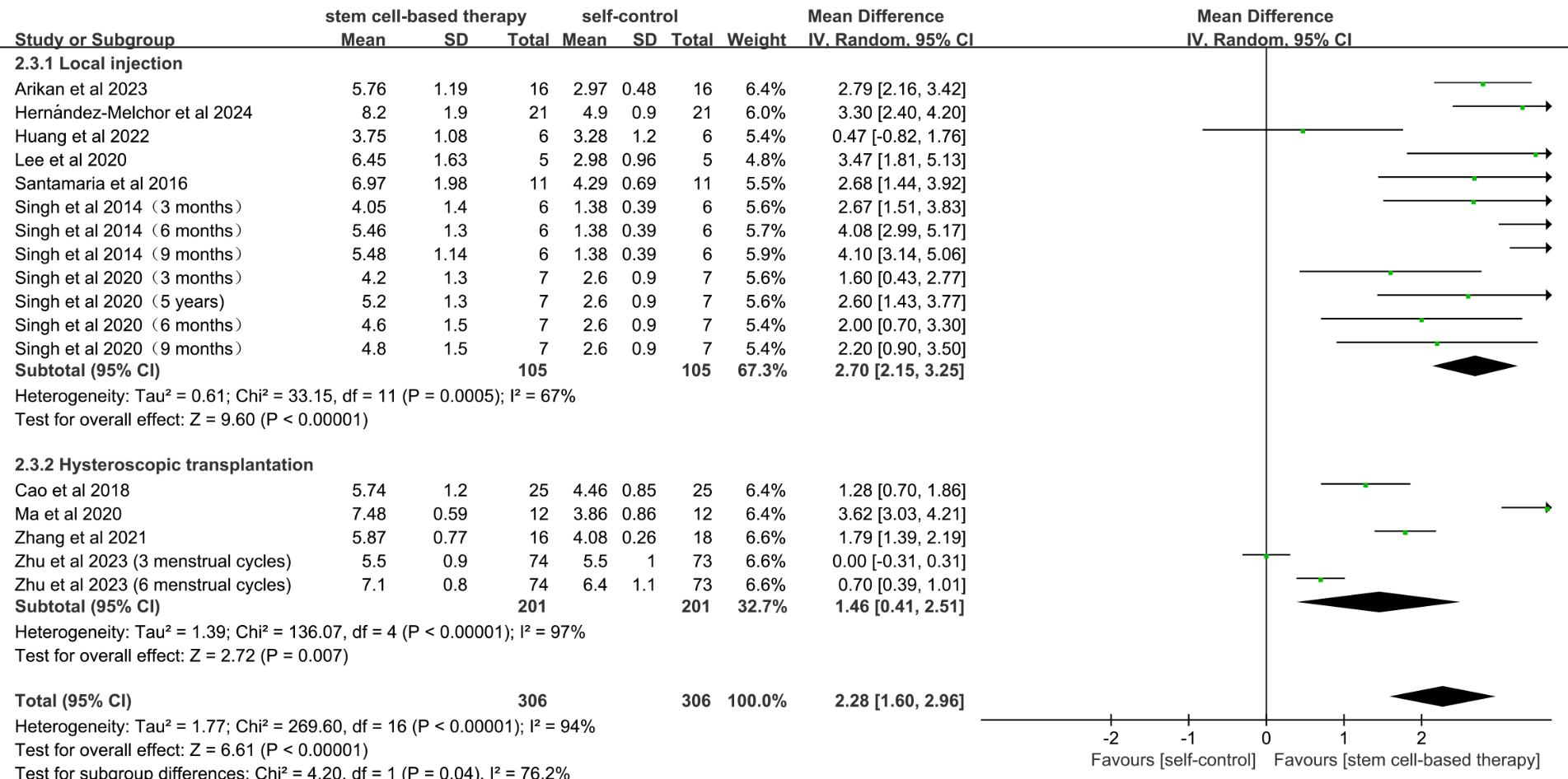


Supplementary Figure 18. Subgroup analysis of endometrial thickness improvement with injection methods.


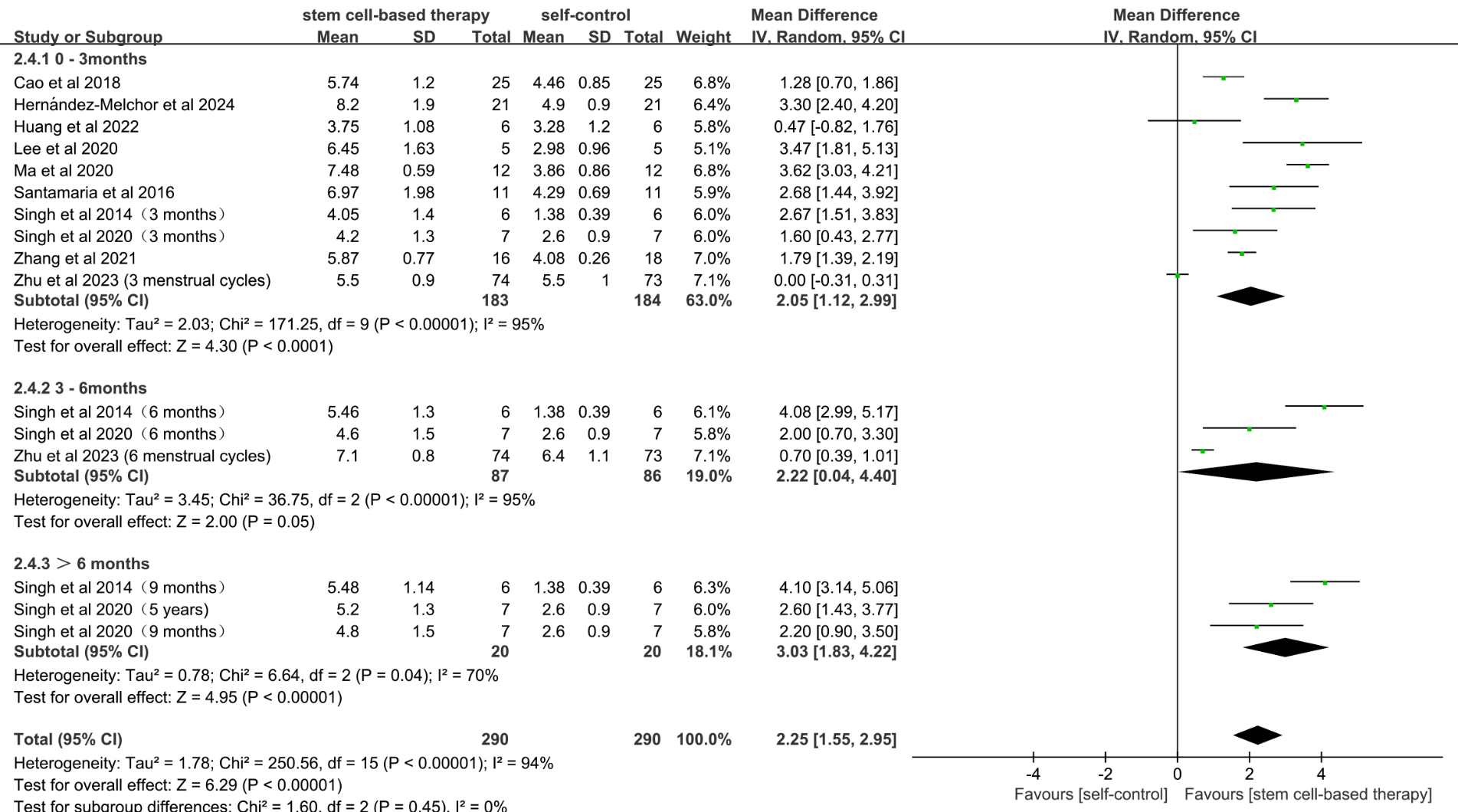


Supplementary Figure 19. Subgroup analysis of endometrial improvement at different times.
